# Association of analgesics prescription with incident comorbidities in people with osteoarthritis—a UK primary care database cohort study

**DOI:** 10.1093/rap/rkag098

**Published:** 2026-08-07

**Authors:** Subhashisa Swain, Carol Coupland, Andrea Dell‘ Isola, Danielle E Robinson, Anne Kamps, Marta Pineda Moncusi, Jos Runhaar, Christian Mallen, Chang Fu Kuo, Daniel Prieto-Alhambra, Martin Englund, Sita Bierma-Zeinstra, Michael Doherty, Weiya Zhang

**Affiliations:** Injury, Recovery and Inflammation Sciences, School of Medicine, University of Nottingham, Nottingham, UK; Nuffield Department of Primary Care Health Sciences, University of Oxford, Oxford, UK; School of Medicine, Keele University, Keele, UK; Centre for Academic Primary Care, School of Medicine, University of Nottingham, Nottingham, UK; Wolfson Institute of Population Health, Queen Mary University of London, London, UK; Clinical Epidemiology Unit, Orthopedics, Department of Clinical Sciences Lund, Lund University, Lund, Sweden; Centre for Statistics in Medicine, Nuffield Department of Orthopaedics, Rheumatology and Musculoskeletal Sciences, University of Oxford, Oxford, UK; Department of General Practice, Erasmus MC University Medical Center Rotterdam, Rotterdam, The Netherlands; Centre for Statistics in Medicine, Nuffield Department of Orthopaedics, Rheumatology and Musculoskeletal Sciences, University of Oxford, Oxford, UK; Department of General Practice, Erasmus MC University Medical Center Rotterdam, Rotterdam, The Netherlands; Centre for Academic Primary Care, School of Medicine, University of Nottingham, Nottingham, UK; Division of Rheumatology, Allergy and Immunology, Chang Gung Memorial Hospital, Taipei, Taiwan; Pain Centre Versus Arthritis, University of Nottingham, Nottingham, UK; Centre for Statistics in Medicine, Nuffield Department of Orthopaedics, Rheumatology and Musculoskeletal Sciences, University of Oxford, Oxford, UK; Clinical Epidemiology Unit, Orthopedics, Department of Clinical Sciences Lund, Lund University, Lund, Sweden; Department of General Practice, Erasmus MC University Medical Center Rotterdam, Rotterdam, The Netherlands; Department of General Practice, Department of Orthopedic Surgery and Sports Medicine, Erasmus MC University Medical Center Rotterdam, Rotterdam, The Netherlands; Injury, Recovery and Inflammation Sciences, School of Medicine, University of Nottingham, Nottingham, UK; Pain Centre Versus Arthritis, University of Nottingham, Nottingham, UK; Injury, Recovery and Inflammation Sciences, School of Medicine, University of Nottingham, Nottingham, UK; Pain Centre Versus Arthritis, University of Nottingham, Nottingham, UK

**Keywords:** osteoarthritis, pain, medication, analgesics, multimorbidity, comorbidity, primary care, health records, opioids, NSAIDs, paracetamol

## Abstract

**Objective:**

We examined the independent risk of incident comorbidities associated with prescribed oral paracetamol, NSAIDs and opioids and their interaction and mediation with OA.

**Methods:**

Exposure of these analgesics and the association with nine systemic groups of incident comorbidities were examined in this 5-year cohort study of 261 273 people with incident OA and 261 273 age-, sex- and practice-matched controls (no OA) in the large UK primary care database. A propensity score–adjusted time-varying exposure analysis was undertaken using multivariable Cox models to estimate the hazard ratios (HRs) for incident comorbidities associated with OA and analgesics. Interaction was defined when the combined term for OA and analgesic was significant. The proportion of the risk associated with OA mediated by analgesics was also calculated.

**Results:**

The mean age of the cohort was 60 years and 57.7% were female. NSAIDs and opioids were independently associated with all comorbidities. Paracetamol interacted with OA leading to an extra risk of renal comorbidities [multiplicative HR (mHR) 1.14 (95% CI 1.06, 1.22)]. NSAIDs and opioids interacted with OA with extra risk of cardiovascular [mHR 1.05 (95% CI 1.01, 1.11) and mHR 1.06 (95% CI 1.02, 1.11)] and endocrine [mHR 1.11 (95% CI 1.05, 1.17) and mHR 1.08 (95% CI 1.02, 1.13)] comorbidities. About 16% and 7% risk from OA to psychological comorbidities were indirectly mediated by NSAIDs and opioids, respectively.

**Conclusion:**

Our findings suggest that in people with OA, analgesic prescriptions may be associated with increased risk of certain incident comorbidities, particularly in long-term use.

Key messages

- Paracetamol, ibuprofen and opioids were linked to a higher risk of heart, hormone, kidney and mental health conditions.
- In >500 000 people, 7–16% of mental health risk was linked to these medicines.
- These medicines added small extra risks beyond osteoarthritis alone, including about 5–11% higher risk for some conditions.

## Introduction

Nearly 8.5 million people in the UK and 10% of patients visiting primary care have OA [1, 2]. People with OA are at increased risk of developing other chronic conditions [3, 4]. Explanations for the association between OA and a large number of chronic conditions vary from direct effects from the low-grade inflammatory process in OA to potential indirect effects from reduced physical activity, increasing the chances of being obese and/or analgesic use. However, the biological explanations for each of the factors needs further investigation. The presence of additional conditions with OA increases the complexity of management and predisposes to polypharmacy, which associates with a decline in quality of care and adherence to management [5].

Paracetamol, NSAIDs and opioids are the three most commonly used analgesics for chronic pain management in people with OA [6, 7]. The annual cost of NSAID prescriptions in people with OA in the UK in 2010 was estimated to be £25.65 million and the cost of NSAID-related iatrogenic events was estimated to be £56.9–£124.4 million in 2010 [8, 9]. Understanding the contribution of OA and the prescribed medicines towards development of other chronic conditions should help optimise management planning.

It is well known that oral paracetamol, NSAIDs and opioid analgesics are associated with increased risk of gastrointestinal, cardiovascular and renal events [10–12]. However, whether they are associated with the development of OA comorbidity and how they contribute to these comorbidities remains unknown. This is essential, as it will help optimise the use of analgesics in the treatment of OA.

We therefore undertook this cohort study comparing the risks of developing multiple long-term conditions in people with *vs* without OA who were prescribed *vs* not prescribed analgesics. The study was undertaken in a large national database of primary healthcare records.

## Methods

For this study we used the GOLD version of the Clinical Practice Research Datalink (CPRD), one of the largest anonymised primary care registry databases in the UK [13]. The study was approved by the Independent Scientific Advisory Committee for CPRD research (protocol reference 19_030 R).

### Study design

We used a matched cohort study design in people with and without a diagnosis of OA to estimate the associations between the three prescribed types of oral analgesics and different comorbidities that developed after the diagnosis date of OA or the equivalent date in matched controls.

### Participants

Individuals ≥20 years of age at the time of OA diagnosis within the study time period, in an up-to-standard (UTS) practice, with data of acceptable quality (determined by the CPRD), and an active registration period of at least 1 year during the period 1 January 1997–31 December 2019 were eligible for inclusion. Hip, knee, ankle, foot, wrist, hand and unspecified OA diagnosis were identified using Read codes (N05). The date of first diagnosis of OA was used as the index date for follow-up. For each person with OA, age- (±2 years), sex- and practice-matched controls without any record of OA or joint replacement were identified in a 1:1 ratio from the same database. Controls were assigned the same index date as their matched person with OA.

### Exposure

**Figure 1:**
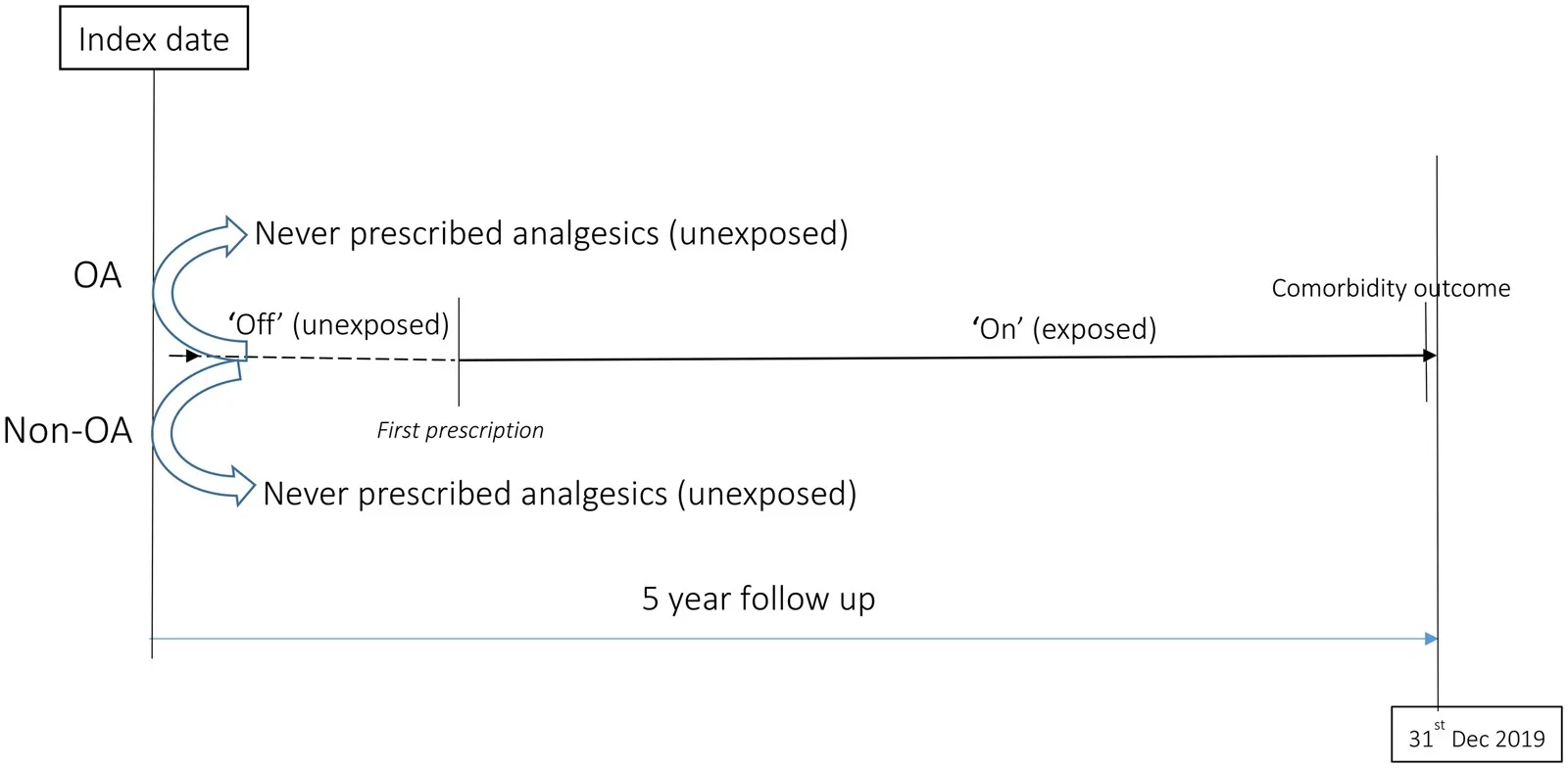
Study design

The prescription data for the three types of analgesics (specifically paracetamol, NSAIDs and opioids) were derived from the ‘therapy’ file of the CPRD using British National Formulary (BNF) codes (Supplementary Table S2.1). For each type of analgesic, anyone with a minimum of two prescriptions within 90 days were eligible for our study [14] and the first prescription was considered as the ‘exposed date’. Exposure to each analgesic was treated as a time-varying variable whereby follow-up time from the index date until the first prescription of the analgesic (i.e. the exposed date) was considered as ‘unexposed’ and the remaining follow-up time was categorised as ‘exposed’. Participants who had never been prescribed analgesics during the follow-up were also considered as ‘unexposed’ (Fig. 1). The main results reported in this study are for the risks associated with each analgesic exposure up to 5 years after the index date.

### Outcomes

For the primary analysis, we grouped the comorbidities into nine disease groups, specifically cardiovascular disease (CVD), gastrointestinal (GI), endocrine and metabolic, psychological, renal, hepatic (liver), oncologic (cancer), neurological and respiratory. People with these outcomes reported before the index date were not included in the analysis of the respective outcome to avoid confounding by indication. Details of the conditions included in each group are given in Supplementary Table S1.1. Groupings of the conditions were made to improve feasibility and reduce the risk of overfitting rather than to imply that all conditions within a category share similar mechanisms or risks.

### Covariates

Baseline characteristics and the comorbidities collected at the index date included age at OA diagnosis/index date, sex, BMI, smoking status and alcohol use. BMI (kg/m^2^) was categorised into the following five groups: underweight (<18.50), normal (18.50–24.99), overweight (25.00–29.99), obese (>30.00) and missing [15]. Smoking status was categorised as non-smoker, ex-smoker, current smoker or missing. Alcohol use was grouped into non-user, ex-user, current user 1–9 units/week, current user >10 units/week, current user (unknown quantity) or missing. The missing categories were used as a separate group in the variable during the statistical analysis.

### Statistical analyses

Analyses were carried out using Stata v18 (StataCorp, College Station, TX, USA) and R v3.5 (R Foundation for Statistical Computing, Vienna, Austria). We analysed exposures for the different analgesics separately. A propensity score–adjusted time-varying exposure (analgesics) analysis was undertaken using a multivariable Cox model to estimate adjusted hazard ratios (aHRs) and 95% CIs for each of the nine comorbidity outcomes [16]. In the main analysis, participants were followed up from the index date until the earliest of the outcome diagnosis, death, 5 years after the index date or the end of the study (31 December 2019). Details about propensity score calculation and performance are provided in Supplementary Tables S1.2 and S1.3 and Supplementary Figs S1.1 and S1.2.

Participants were stratified into four strata according to initial prescription status with each analgesic (D) separately at exposure period: ‘No OA and no drugs’ (OA^−^D^−^), ‘OA and no drugs’ (OA^+^D^−^), ‘No OA and drugs’ (OA^−^D^+^) and ‘OA and drugs’ (OA^+^D^+^). For example, an individual could belong to the OA^−^D^−^ group at the index date and move to the OA^−^D^+^ group after the prescription date (Fig. 1). Unadjusted and adjusted HRs and 95% CIs were calculated for all groups against the reference group (OA^−^D^−^). The proportional hazards assumption was tested using the Schoenfeld test. In the adjusted model, the estimated propensity score was adjusted for. Cumulative hazards and incidence rates (per 1000 person-years) were reported for each comorbidity outcome. The multiple testing problem was addressed using the false discovery rate Benjamini–Hochberg approach (FDR package in R).

Multiplicative interactions between OA and analgesic exposure for each incident comorbidity were estimated using the HR by including an interaction term (OA*Drug) in the final adjusted model. Additive interactions were calculated for outcomes with positive multiplicative interactions using three indicators proposed by Rothman *et al*. [17]. We reported relative excessive risk due to interaction (RERI), attributable proportion (AP) and synergy index (SI), each with respective 95% CIs. Details of methods of calculation are given in Supplementary Table S1.4. If the RERI and AP are equal to 0 and SI is equal to 1, that suggests an absence of additive interactions. RERI >0, AP >0 and SI >1 implies a synergetic interaction, i.e. the combined action between two exposures in the additive model is greater than the sum of the individual effects. However, if RERI <0, AP <0 and SI <1, it implies an antagonistic interaction, meaning that in the presence of two exposures in an additive model, the action of one exposure diminishes the effect of the other [17].

We also performed mediation analyses in Stata using the ‘med4way’ package to explore the proportion of the association between OA and comorbidities mediated by analgesics. Total natural direct effect (TNDE) and total natural indirect effect (TNIE) were estimated in an adjusted model to explain the mediator role. TNIE represented the effect of OA on comorbidities in the presence of mediators (analgesics) and TNDE represented the effect without the mediators (i.e. no analgesics). The proportion of association by the mediator was reported to quantify the magnitude of the mediation [18].

The dose–response relationship was examined by first calculating the total number of prescriptions made between the index date and the date of the end of follow-up or death/left practice if earlier and then dividing the total number of prescriptions by the number of follow-up years. We used the quintile of the derived prescription score Q1 (lowest) to Q5 (highest).

### Sensitivity analyses

For the sensitivity analyses we repeated the analysis for 1, 10 and the maximum of 22 years follow-up from the index date using the same methods. We also examined the effects of NSAIDs co-prescribed with proton pump inhibitors (PPIs) on the risk of incident comorbidities (Supplementary Table S2.2).

### Patient and public involvement and engagement

Three patients from three different European countries were involved from the very beginning of the study, providing essential input at every stage of the research process. They were actively engaged in discussions, helping shape the study design and offering valuable perspectives on the analysis and interpretation of the data. Their feedback was incorporated into the final stages of the research, ensuring that the findings were both patient-centred and clinically relevant. The results were shared with the patient group for their review and their suggestions were thoughtfully integrated when finalizing the manuscript.

## Results

During the study period, we identified 261 273 people with incident OA and an equal number of sex-, age- and practice-matched controls. The mean age in the OA and control groups was 59.5 years (s.d. 12.8) and 60.3 years (s.d. 12.9), respectively, with 57.7% women in both groups. The prevalence of obesity at the index date was 32% in the OA group compared with 22% in the non-OA group. Of the three studied analgesics, opioids were the most commonly prescribed during follow-up (OA 64%, non-OA 38%), followed by NSAIDs (OA 57%, non-OA 30%) and paracetamol (OA 29%, non-OA 16%). The median length of follow-up was 4.8 years [interquartile range (IQR) 2.0–8.8] in the OA group and 6.4 years (IQR 3.0–10.8) in controls. Details of descriptive statistics are provided in Table 1. Description of the study population by exposure groups is provided in Supplementary Table S1.5.

**Table 1:** Demographic characteristics of study participants at the index date.

Characteristics	Incident OA (*n* = 261 273)	Non-OA (*n* = 261 273)
Age, years, mean (s.d.)	59.47 (12.83)	60.26 (12.88)
Age in men, years, mean (s.d.)	59.44 (12.57)	60.23 (12.60)
Age in women, years, mean (s.d.)	59.47 (13.02)	60.28 (13.08)
BMI, mean (s.d.)	28.34 (5.67)	26.76 (5.06)
Age at index date (years), *n* (%)		
<40	17 126 (6.55)	15 478 (5.92)
40–49	41 836 (16.01)	38 734 (14.83)
50–59	77 741 (29.75)	76 274 (29.19)
60–69	68 652 (26.28)	70 705 (29.19)
70–79	40 542 (15.52)	42 823 (16.39)
80–89	14 362 (5.5)	15 410 (5.9)
≥90	1014 (0.39)	1849 (0.71)
Gender, *n* (%)		
Male	110 414 (42.26)	110 414 (42.26)
Female	150 859 (57.74)	150 859 (57.74)
BMI (kg/m^2^), *n* (%)		
<18.5 (underweight)	3438 (1.32)	5371 (2.06)
18.5–24.9 (normal)	72 184 (27.63)	96 143 (36.8)
25.0–29.9 (overweight)	92 824 (35.53)	94 024 (35.99)
≥30 (obese)	83 800 (32.07)	56 569 (21.65)
Missing	9027 (3.46)	9166 (3.51)
Alcohol consumption (units/week), *n* (%)		
Never	47 799 (18.29)	45 881 (17.56)
Ex-drinker	6676 (2.56)	6091 (2.33)
Current 1–9	87 750 (33.59)	88 963 (34.05)
Current ≥10	49 704 (19.02)	49 175 (18.82)
Current unknown	57 888 (22.16)	58 674 (22.46)
Missing	11 456 (4.38)	12 489 (4.78)
Smoking status, *n* (%)		
Never smoked	138 223 (52.9)	143 844 (55.06)
Ex-smoker	72 132 (27.61)	68 274 (26.13)
Current smoker	50 381 (19.28)	48 632 (18.61)
Missing	537 (0.21)	523 (0.2)
Analgesic prescription at least once ever, *n* (%)		
Paracetamol	76 474 (29.27)	41 620 (15.93)
NSAIDs	149 566 (57.25)	78 595 (30.08)
Opioids	166 203 (63.61)	99 882 (38.23)
PPI	172 384 (65.98)	136 180 (52.12)
Comorbidities at index date, *n* (%)		
Cancer	12 320 (4.72)	12 369 (4.73)
Cardiovascular	97 073 (37.15)	85 986 (32.91)
Endocrine	65 209 (24.96)	55 834 (21.37)
Gastrointestinal	41 058 (15.71)	32 058 (12.27)
Renal	14 054 (5.38)	12 878 (4.93)
Liver	17 214 (6.59)	13 108 (5.02)
Neurological	44 634 (17.08)	39 345 (15.06)
Psychological	80 371 (30.76)	66 166 (25.32)
Respiratory	49 939 (19.11)	39 745 (15.21)

### Association of comorbidities during 5 years of follow-up with analgesic exposures

Of the four groups (OA^−^D^−^, OA^+^D^−^, OA^−^D^+^ and OA^+^D^+^) studied, the unadjusted incidence rates of comorbidities except for renal were the highest for OA^+^D^+^ in paracetamol users (Supplementary Table S1.6).

Among NSAID users, the OA^−^D^+^ group had increased risks toward incident comorbidity that ranged from 16% to 54% and in the OA^+^D^+^ group it ranged from 18% to 73%. The leading comorbidities in the OA^+^D^+^ group were GI, psychological, cancer and respiratory comorbidities (Table 2). In the opioid users, the OA^−^D^+^ group had an increased risk of diagnosis of comorbidities ranging from 10% to 55% and the high risk was found for diagnosis of liver, renal, GI and psychological comorbidities, whereas in the OA^+^D^+^ group the risk ranged from 17% to 57% higher compared with the OA^−^D^−^ group towards incident psychological, liver and GI comorbidities (Table 2). The increased risk of incident comorbidities in the paracetamol OA^+^D^+^ group compared with the OA^−^D^−^ group ranged from 12% to 53%. The top five conditions associated with OA^+^D^+^ were GI, psychological, liver, cancer and renal (Table 3). A dose–response relationship across the quintiles of cumulative average annual numbers of prescriptions was found with all analgesics for the development of all comorbidities (Supplementary Fig. S1.3a–c, Supplementary  Tables S2.7–S2.9) Non-selective and cyclooxygenase-2 selective NSAID users had higher risks of developing CVD, endocrine, renal, and psychological conditions compared with non-users (Supplementary Table S2.10).

**Table 2:** Associations of analgesics with development of comorbidities in multivariable time-varying Cox models.

Conditions	OA	Drug	Paracetamol		NSAIDs		Opioids	
			*n* (%)	aHR^a^ (95% CI)	*n* (%)	aHR^a^ (95% CI)	n (%)	aHR^a^ (95% CI)
Cancer	−	−	217 599 (43.71)	1	207 975 (41.77)	1	178 440 (35.84)	1
	+	−	189 017 (37.97)	**1.09 (1.06, 1.13)**	156 328 (31.40)	**1.25 (1.20, 1.30)**	119 513 (24.01)	**1.10 (1.06, 1.15)**
	−	+	31 305 (6.29)	0.93 (0.84, 1.02)	40 929 (8.22)	**1.26 (1.15, 1.37)**	70 464 (14.15)	**1.22 (1.14, 1.31)**
	+	+	59 936 (12.04)	**1.30 (1.22, 1.38)**	92 625 (18.60)	**1.42 (1.35, 1.50)**	129 440 (26.00)	**1.40 (1.33, 1.47)**
Cardiovascular	−	−	162 225 (47.79)	1	148 473 (43.73)	1	138 067 (40.67)	1
	+	−	137 554 (40.52)	1.02 (1.00, 1.04)	104 358 (30.74)	1.01 (0.99, 1.03)	93 318 (27.49)	1.01 (0.99, 1.03)
	−	+	13 062 (3.85)	0.98 (0.92, 1.05)	26 814 (7.90)	**1.21 (1.16, 1.27)**	37 220 (10.96)	**1.19 (1.14, 1.24)**
	+	+	26 646 (7.85)	**1.17 (1.12, 1.22)**	59 842 (17.63)	**1.29 (1.26, 1.33)**	70 882 (20.88)	**1.28 (1.24, 1.32)**
Endocrine	−	−	184 121 (45.86)	1	174 059 (43.35)	1	154 740 (38.54)	1
	+	−	156 099 (38.88)	**1.06 (1.04, 1.08)**	125 213 (31.19)	1.00 (0.98, 1.03)	103 099 (25.68)	1.02 (1.01, 1.04)
	−	+	21 318 (5.31)	0.96 (0.89, 1.02)	31 380 (7.82)	**1.17 (1.11, 1.23)**	50 699 (12.63)	**1.10 (1.06, 1.15)**
	+	+	39 965 (9.95)	**1.12 (1.08, 1.17)**	70 851 (17.65)	**1.31 (1.27, 1.35)**	92 965 (23.15)	**1.22 (1.18, 1.26)**
Gastrointestinal	−	−	200 647 (44.64)	1	192 164 (42.76)	1	165 948 (36.92)	1
	+	−	168 041 (37.39)	**1.34 (1.30, 1.39)**	138 059 (30.72)	**1.26 (1.21, 1.31)**	108 017 (24.03)	**1.24 (1.20, 1.29)**
	−	+	28 568 (6.36)	**1.21 (1.10, 1.33)**	37 051 (8.24)	**1.54 (1.42, 1.67)**	63 267 (14.08)	**1.36 (1.27, 1.45)**
	+	+	51 174 (11.61)	**1.53 (1.43, 1.63)**	82 156 (18.28)	**1.73 (1.64, 1.82)**	122 198 (24.96)	**1.54 (1.46, 1.61)**
Liver	−	−	216 196 (43.92)	1	207 468 (42.15)	1	177 023 (35.96)	1
	+	−	184 866 (37.56)	**1.22 (1.17, 1.28)**	153 368 (31.16)	**1.12 (1.08, 1.18)**	117 078 (23.79)	**1.13 (1.08, 1.19)**
	−	+	31 969 (6.49)	**1.28 (1.13, 1.45)**	40 697 (8.27)	**1.18 (1.06, 1.32)**	71 142 (14.45)	**1.55 (1.42, 1.68)**
	+	+	59 193 (12.03)	**1.40 (1.29, 1.53)**	90 691 (18.42)	**1.28 (1.19, 1.37)**	126 981 (25.80)	**1.55 (1.45, 1.65)**
Neurological	−	−	196 296 (44.76)	1	186 182 (42.25)	1	162 095 (36.96)	1
	+	−	166 557 (37.98)	1.02 (0.99, 1.04)	136 845 (31.20)	**1.08 (1.04, 1.11)**	106 734 (24.34)	1.01 (0.98, 1.04)
	−	+	25 632 (5.84)	**1.27 (1.18, 1.36)**	35 746 (8.15)	**1.16 (1.08, 1.25)**	59 833 (13.64)	**1.21 (1.14, 1.28)**
	+	+	50 082 (11.42)	**1.24 (1.18, 1.30)**	79 794 (18.19)	**1.24 (1.19, 1.30)**	109 905 (25.06)	**1.17 (1.12, 1.22)**
Psychological	−	−	171 864 (45.71)	1	166 000 (44.15)	1	144 609 (38.46)	1
	+	−	140 139 (37.27)	**1.31 (1.28, 1.35)**	117 671 (31.29)	**1.11 (1.08, 1.14)**	93 732 (24.93)	**1.18 (1.15, 1.22)**
	−	+	23 243 (6.18)	**1.18 (1.08, 1.28)**	29 107 (7.74)	**1.24 (1.16, 1.33)**	50 498 (13.43)	**1.33 (1.26, 1.40)**
	+	+	40 763 (10.84)	**1.52 (1.44, 1.60)**	63 231 (16.82)	**1.49 (1.44, 1.56)**	87 170 (23.18)	**1.57 (1.51, 1.63)**
Renal	−	−	219 384 (44.27)	1	207 571 (41.88)	1	180 366 (36.39)	1
	+	−	189 767 (38.29)	**0.84 (0.82, 0.87)**	254 243 (31.12)	0.98 (0.95, 1.00)	120 050 (24.22)	**0.84 (0.81, 0.86)**
	−	+	29 011 (5.85)	**1.30 (1.23, 1.40)**	40 824 (8.24)	**1.28 (1.21, 1.36)**	68 029 (13.73)	**1.45 (1.39, 1.52)**
	+	+	57 452 (11.59)	**1.27 (1.21, 1.32)**	92 976 (18.76)	**1.18 (1.13, 1.23)**	127 169 (25.66)	**1.28 (1.23, 1.32)**
Respiratory	−	−	194 324 (44.89)	1	185 551 (42.87)	1	16 0936 (37.18)	1
	+	−	162 050 (37.44)	**1.11 (1.08, 1.16)**	132 961 (30.72)	1.06 (1.02, 1.10)	104 847 (24.22)	1.02 (0.99, 1.06)
	−	+	27 204 (6.28)	**1.21 (1.10, 1.32)**	35 977 (8.31)	**1.19 (1.09, 1.30)**	60 592 (14.00)	**1.26 (1.19, 1.35)**
	+	+	49 284 (11.39)	**1.24 (1.16, 1.32)**	78 373 (18.11)	**1.33 (1.27, 1.41)**	10 6487 (24.60)	**1.28 (1.22, 1.34)**

a Adjusted for age at index date, sex, smoking, alcohol, propensity score, other comorbidities at index date and other analgesics.

Bold indicates significant at *P* < 0.05 after adjusting for multiple testing.

**Table 3:** Both multiplicative and additive interaction between OA and three studied analgesics for comorbidities.

Outcome		Paracetamol, estimate (95% CI)	NSAIDs, estimate (95% CI)	Opioids, estimate (95% CI)
Cardiovascular	mHR	**1.16 (1.07, 1.22)**	**1.05 (1.01, 1.11)**	**1.06 (1.02–1.11)**
	RERI	0.17 (0.13, 0.20)	**0.07 (0.03, 0.11)**	**0.08 (0.05, 0.11)**
	AP	0.21 (0.17, 0.26)	**0.05 (0.02, 0.08)**	**0.06 (0.04, 0.09)**
	Synergy index	0 (−1.5, 2.4)	**1.31 (1.10, 1.73)**	**1.40 (1.18, 1.84)**
Cancer	mHR	**1.27 (1.14, 1.32)**	0.90 (0.81, 1.00)	1.03 (0.96, 1.12)
	RERI	0.28 (0.23, 0.32)	−0.09 (−0.11, −0.06)	**0.08 (0.01, 0.13)**
	AP	0.21 (0.17, 0.26)	−0.06 (−0.11, 0.0)	**0.05 (0.01, 0.09)**
	Synergy index	15 (−2.22, 25.30)	0.82 (0.74, 1.00)	**1.25 (1.02, 1.65)**
Endocrine	mHR	**1.10 (1.02, 1.19)**	**1.11 (1.05,1.17)**	**1.08 (1.03, 1.13)**
	RERI	0.10 (0.07, 0.15)	**0.14 (0.09, 0.18)**	**0.10 (0.07, 0.11)**
	AP	0.08 (0.06, 0.14)	**0.11 (0.07, 0.14)**	**0.08 (0.05, 0.09)**
	Synergy index	6.0 (−1.10, 17.0)	**1.82 (1.34, 3.00)**	**1.83 (1.36, 2.57)**
Gastrointestinal	mHR	0.94 (0.84, 1.05)	**0.88 (0.81, 0.97)**	**0.90 (0.84, 0.99)**
	RERI	−0.02 (−0.09, 0.03)	−0.07 (−0.16, 0.01)	**−0.06 (−0.13, −0.01)**
	AP	−0.01 (−0.05, 0.02)	−0.04 (−0.08, 0.01)	−0.04 (−0.08, 0.00)
	Synergy index	0.96 (0.87, 1.07)	0.91 (0.84, 1.01)	**0.90 (0.82, 0.98)**
Liver	mHR	0.89 (0.78, 1.03)	**0.83 (0.72, 0.95)**	**0.80 (0.79, 0.97)**
	RERI	−0.10 (−0.20, −0.10)	−0.02 (−0.13, 0.05)	**−0.13 (−0.22, −0.05)**
	AP	−0.07 (−0.13, −0.01)	−0.01 (−0.09, 0.04)	**−0.08 (−0.13, −0.03)**
	Synergy index	0.80 (0.72, 0.96)	0.93 (0.74, 1.35)	**0.80 (0.74, 0.90)**
Neurological	mHR	0.95 (0.88, 1.04)	0.98 (0.90, 1.07)	0.96 (0.90, 1.02)
	RERI	−0.05 (−0.10, 0.01)	0 (−0.06, 0.07)	−0.05 (−0.02, 0.00)
	AP	−0.04 (−0.07, 0.01)	0 (−0.04, 0.06)	−0.04 (−0.08, 0.00)
	Synergy index	0.83 (0.75, 1.06)	1.00 (0.83, 1.58)	0.77 (−0.68, 1.00)
Psychological	mHR	0.98 (0.89, 1.07)	**1.08 (1.01, 1.16)**	0.99 (0.93, 1.06)
	RERI	0.03 (−0.03, 0.08)	**0.14 (0.09, 0.20)**	**0.06 (0.01, 0.10)**
	AP	0.02 (−0.01, 0.05)	**0.09 (0.05, 0.14)**	**0.04 (0.01, 0.07)**
	Synergy index	1.06 (0.95, 1.22)	**1.40 (1.19, 1.83)**	**1.11 (1.02, 1.24)**
Renal	mHR	**1.14 (1.06, 1.22)**	0.94 (0.88, 1.01)	1.04 (0.98, 1.10)
	RERI	**0.13 (0.05, 0.16)**	−0.08 (−0.13, −0.03)	−0.01 (−0.06, 0.03)
	AP	**0.10 (0.03, 0.13)**	−0.06 (−0.10, −0.02)	−0.01 (−0.04, 0.02)
	Synergy index	**1.93 (1.18, 4.20)**	0.69 (0.63, 0.81)	0.96 (0.84, 1.15)
Respiratory	mHR	0.91 (0.82, 1.02)	1.05 (0.95 − 1.16)	0.99 (0.92, 1.07)
	RERI	−0.08 (−0.16, −0.02)	**0.08 (0.01, 0.16)**	0 (−0.07, 0.04)
	AP	−0.06 (−0.12, −0.01)	**0.06 (0.01, 0.12)**	0 (−0.05, 0.03)
	Synergy index	0.75 (0.66, 0.88)	**1.32 (1.02, 2.45)**	1.0 (0.83, 1.22)

mHR obtained from the adjusted model with ‘OA*drug’ variable.

Bold indicates significant at *P* < 0.05.

### Interactions between OA and analgesic exposure

For paracetamol users, we found a multiplicative interaction with OA only for cancer, CVD, endocrine and renal comorbidities and a weak additive interaction for renal comorbidities. NSAID users and opioid users had a significant multiplicative interaction for CVD, endocrine, GI, liver and psychological comorbidities and an additive interaction was significant for CVD, endocrine, psychological, cancer and respiratory comorbidities (Table 3).

### Mediation

Among paracetamol users, mediation analysis suggested no relevant mediation role for the drug. NSAIDs had a mediation role for the incident CVD and endocrine conditions contributing 45% (95% CI 18, 72) and 52% (95% CI 23, 82) towards the total risk and 10–20% of risk for new diagnosis of GI, neurological, psychological and respiratory conditions. Prescription of opioids had contributions through a mediation role for incident CVD (35%), cancer (12%), endocrine (18%), liver (16%), psychological (7%) and GI (5%) (Table 4).

**Table 4:** Role of mediation for analgesics in OA for incident comorbidities.

Conditions		Paracetamol, estimate (95% CI)	NSAIDs, estimate (95% CI)	Opioids, estimate (95% CI)
Cardiovascular	NDE	**1.03 (1.01, 1.05)**	1.02 (0.99, 1.04)	1.02 (0.99, 1.05)
	NIE	1.00 (1.00, 1.01)	**1.02 (1.01, 1.03)**	1.01 (1.00, 1.02)
	Proportion mediated (%)	8.8 (3.5, 14.1)	45.2 (18.7, 71.6)	35.3 (16.4, 54.2)
Cancer	NDE	**1.12 (1.07, 1.18)**	**1.26 (1.18, 1.34)**	**1.12 (1.05, 1.18)**
	NIE	1.00 (0.99, 1.01)	1.01 (0.99, 1.03)	1.01 (1.00, 1.02)
	Proportion mediated (%)	4.8 (2.6, 7.1)	5.4 (3.0, 7.8)	11.6 (7.8, 15.4)
Endocrine	NDE	**1.07 (1.04, 1.09)**	1.02 (0.98,1.05)	1.03 (1.00, 1.07)
	NIE	1.00 (0.99, 1.00)	**1.02 (1.01, 1.03)**	1.00 (0.99, 1.01)
	Proportion mediated (%)	1.8 (0.4, 3.3)	52.2 (22.8, 81.6)	18.5 (10.5, 26.7)
Gastrointestinal	NDE	**1.03 (1.02-1.04)**	**1.26 (1.18, 1.33)**	**1.24 (1.17, 1.32)**
	NIE	1.00 (0.99-1.01)	1.03 (1.01, 1.05)	1.01 (1.01, 1.02)
	Proportion mediated (%)	1.18 (0.6, 1.8)	11.8 (9.2, 14.3)	5.4 (3.9, 6.9)
Liver	NDE	**1.23 (1.15, 1.31)**	**1.13 (1.05, 1.21)**	**1.10 (1.02, 1.18)**
	NIE	1.00 (0.99, 1.01)	1.01 (0.90, 1.03)	**1.27 (1.02, 1.35)**
	Proportion mediated (%)	2.2 (0.7, 3.7)	8.4 (2.7, 14.2)	16.5 (9.1, 23.9)
Neurological	NDE	1.02 (0.98, 1.05)	**1.08 (1.03, 1.12)**	1.00 (1.00, 1.03)
	NIE	1.01 (1.00, 1.02)	1.01 (1.00, 1.02)	1.01 (1.01, 1.02)
	Proportion mediated (%)	25.6 (−5.25, 56.4)	12.3 (6.5, 18.1)	100 (−239, 441)
Psychological	NDE	**1.36 (1.31, 1.42)**	**1.12 (1.07, 1.17)**	**1.19 (1.15, 1.26)**
	NIE	1.00 (0.99, 1.01)	**1.02 (1.01, 1.03)**	**1.01 (1.01, 1.02)**
	Proportion mediated (%)	0.9 (0.05, 1.27)	15.9 (11.9, 20.0)	6.9 (5.5, 8.3)
Renal	NDE	0.86 (0.84, 0.89)	0.97 (0.93, 1.01)	0.86 (0.83, 0.89)
	NIE	1.01 (1.00, 1.02)	1.02 (1.00, 1.02)	1.02 (1.01, 1.03)
	Proportion mediated (%)	−10.3 (−12.7, −7.9)	−9.2 (−24.6, 56.2)	−18.3 (−22.5, −14.8)
Respiratory	NDE	**1.11 (1.07, 1.16)**	**1.07 (1.02, 1.13)**	1.02 (0.97, 1.08)
	NIE	1.00 (0.98,1.00)	1.01 (1.00, 1.03)	1.01 (1.00, 1.02)
	Proportion mediated (%)	2.26 (0.7, 3.8)	19.7 (10.4, 29.1)	33.2 (−0.8, 67.3)

NDE: natural direct effect; NIE: natural indirect effect.

Bold indicates significant at P < 0.05.

### Sensitivity analysis

The risks associated with analgesics exposure assessed over 1, 10 and 22 years (maximum) of follow-up are given in Supplementary Tables S2.3–S2.5. The pattern of increasing cumulative hazard of risk of comorbidities is provided in Supplementary Figs S2.1–S2.3. More than 70% of people prescribed with any of the studied analgesics were also prescribed PPIs compared with the non-analgesics group and a reduction in risk associated with NSAIDs exposure after adjusting for PPI use was seen for all the comorbidities, primarily for GI, psychological and endocrine conditions (Supplementary Table S2.6).

## Discussion

This observational study examined the prescribing patterns of oral paracetamol, NSAIDs and opioids and their associations with the incident risk of nine systemic comorbidities in individuals with and without OA within the UK primary care setting. The key findings are nearly two-thirds of people with OA were prescribed opioids and NSAIDs, while just one-third were prescribed paracetamol over a median follow-up period of 4.8 years after OA diagnosis; paracetamol was independently associated with some but NSAIDs and opioids were independently associated with all comorbidities examined; synergistic interactions were observed between OA and analgesic use in relation to the development of various comorbidities; and the mediating role of analgesics was more pronounced for NSAID and opioid use than for paracetamol in the development of certain chronic conditions.

### Drug use proportions and comparisons

According to the current National Institute for Health and Care Excellence guideline, NSAIDs are the first recommended oral analgesic for people with OA and intermittent prescription of paracetamol where possible [7]. In our study only 30% were prescribed paracetamol during follow-up, whereas 57% were prescribed NSAIDs and 64% opioids. However, this may underestimate the actual use of paracetamol and NSAIDs due to their availability over the counter (OTC) in the UK, where only 5.7% of people were prescribed paracetamol [19]. An increasing trend of opioid prescriptions for pain management in OA has been noted around the world [19–21].

### Risk of developing comorbidities

Paracetamol is prescribed free to patients ≥60 years of age in England and is available OTC because of its relative safety features, creating the possibility of ‘indication bias’ in the study. However, the multiplicative interaction for the development of CVD, cancer and endocrine conditions suggests a synergistic effect of OA and paracetamol. The increased incidence risk of six comorbidities associated with paracetamol in our study is perhaps surprising, as previously perceived [22, 23]. Even though Scotland, Wales and Northern Ireland provide free prescriptions for everyone, the comparison with the current findings needs exploring.

The association with different comorbidities and NSAIDs in people with and without OA is in line with previous evidence [24]. The increased risks in the NSAID group for GI bleeding [25], CVD [26] and renal problems [27] in NSAID users in the present study accords with existing evidence.

Increased opioid prescriptions and their effects from long-term use is reported to be associated with multiple comorbidities [27–29], similar to those reported in our study. The observed associations with incident cancer may reflect reverse causation, whereby early, undiagnosed cancer-related symptoms lead to increased analgesic use prior to diagnosis, as well as higher detection rates due to increased healthcare utilisation; these findings should therefore be interpreted with caution. The dose–response associations for all analgesics were surprisingly high in the highest quintile for all studied comorbidities. Although confounding by indication, surveillance bias and reverse causality cannot be ruled out, caution should be exercised when using analgesics to treat OA, as they may increase the risk of other long-term conditions. This indicates that consideration should be given to alternatives to chronic use of analgesics and to optimising non-pharmacological management of pain in primary care.

### Interactions and mediation

We found that initiation of analgesics was associated with incident comorbidities irrespective of having OA, indicating the underlying role of both OA and NSAIDs. Until now, there has been a lack of studies looking at the mediating role of analgesics for incident comorbidities in people with OA. Except for paracetamol, nearly 45–50% of the total risk for CVD and endocrine comorbidities were explained through NSAIDs, similar to findings reported in Canada [30]. The nearly 20% of risk contribution from NSAIDs for psychological and respiratory conditions needs further investigation. Similarly, the independent harmful effect of opioids cannot be completely ruled out.

We defined exposure as at least two analgesic prescriptions within 6 months, which indirectly indicates the severity and chronicity of OA in the study population. The severity and chronicity often cause decreased physical activity and an increasing risk of obesity. Although we adjusted for BMI in our analysis, the role of obesity and lack of physical activity cannot be completely ruled out. Analgesic prescribing may partly reflect underlying disease severity and symptom burden and therefore the observed mediation effects should be interpreted with caution as they may represent, in part, confounding by indication rather than a direct causal pathway. Diagnostic surveillance bias may also have contributed, as individuals receiving regular prescriptions are likely to have more frequent healthcare contacts, increasing opportunities for comorbidity detection.

### Future direction

People with OA requiring analgesic management may thus be exposed to a higher risk of new comorbidities, which not only depends on the pharmacological effect of the drug but also the underlying condition for which the drug is prescribed. This demands careful consideration of benefits and potential risks when these medicines are prescribed, and long-term monitoring of people prescribed these analgesics irrespective of whether they have OA. Future studies should explore mediation effects using more focused models and robust causal inference frameworks, especially towards psychological conditions.

### Strengths and limitations

Strengths of this study include a representative well-characterised cohort of people with and without OA, matched controls, incident sampling, use of propensity scores to balance the groups, a large sample size for assessing interactions, mediation calculation, long follow-up time (22 years maximum) and adjustment for multiple testing. However, there are a number of important caveats to consider. Propensity score use was not estimated for multiple time-varying exposures. The prescribed drugs that are recorded do not necessarily reflect the medication consumption by the people. Our outcome was grouped as per the body systems and could have included different conditions with different pathologies involved within the same grouping (e.g. renal). Another limitation is that the OTC use of drugs such as paracetamol and low-dose NSAIDs is not recorded in the database. There is always a risk of ascertainment bias in recording of the conditions, causing underestimation of the comorbidity burden. Our method minimised the immortal time bias during the non-exposure periods, but this cannot consider the carryover effects of analgesics, future changes in exposure and the long-term chronic damage of analgesics. Even though this study does not compare the three analgesics, there is the possibility of having confounding by indication, since more frail people possibly would have been prescribed paracetamol in preference to other analgesics. We did not study the impact of stopping or switching drugs and the potential benefits on comorbidity. People in our study might have had multiple drug type prescriptions given at the same time, but we focused on the association of each main exposure adjusting for other prescriptions. We could not adjust for lifestyle factors such as diet and physical activity because such data are not available in the CPRD, so there is likely to be some residual confounding.

## Conclusion

We have found that people with OA are at higher risk of having comorbidities than those without OA. Analgesics may contribute to this risk either directly or indirectly (as a mediator). As this is an observational study, the findings are of associations rather than causal effects. The dataset is large and broadly representative, but lack of data on pain severity, functional status, OTC use and frailty limits causal inference. Future research is required to identify the latent factors predisposing to the risk of each comorbidity.

## Supplementary Material

rkag098_Supplementary_Data

## Data Availability

The data underlying this article are available in CPRD, at https://doi.org/10.11581/d86m-gd03. The datasets were derived from sources in the public domain: https://commons.datacite.org/ror.org/01h3bmp72 obtainable via appropriate approval.
