# Supplementary material for: Association of analgesics prescription with incident comorbidities in people with osteoarthritis—a UK primary care database cohort study
Source: Rheumatol Adv Pract. 2026 Aug 7;10(3):rkag098. doi: 10.1093/rap/rkag098 (PMC13529369; doi:10.1093/rap/rkag098)
Supplement: Supplementary file 1 [file rkag098_supplementary_data.zip › Analgesic_Supplementary File1.docx]

**Supplementary file 1**

Contents

[Comorbidity selection	2](#_Toc211542634)

[Study methods	3](#_Toc211542635)

[Table S1.1 List of the conditions and codes used in the study	4](#_Toc211542636)

[Propensity score calculation	6](#_Toc211542637)

[Table S1.2 Covariates included in propensity score estimation	6](#_Toc211542638)

[Figure S1.1 Estimated propensity scores for three types of analgesics	7](#_Toc211542639)

[Table S1.3 Comparison of model performance after weight calculation	7](#_Toc211542640)

[Figure S1.2 Change in KS score before and after PS weighting	8](#_Toc211542641)

[Table S1.4 Notation and measures additive interaction calculation	9](#_Toc211542642)

[Table S1.5 Descriptive statistics of the study population across exposure groups	10](#_Toc211542643)

[Table S1.6 Incidence rates of comorbidities per 1000 person-years over five years after the index date across different groups for three analgesics	11](#_Toc211542644)

[Figure S1.3. Dose-response association of analgesics in OA, non-OA, and total	13](#_Toc211542645)

# Comorbidity selection

We defined comorbidity as the recording of the diagnosis of predefined chronic conditions in individuals of both groups at any time in the database. An extensive list of 61 chronic conditions was prepared from the Quality Outcome Framework (QOF) (1), list of the US Department of Health and Human Services Initiative on Multiple Chronic Conditions (2), and the Charlson comorbidity index (3). The list was updated with findings from our systematic review (4) and a previous UK community-based knee pain study (4,5) by including common and important morbidities not included in the above (6,7). As long as they were available from the Read codes in CPRD, they were included.

The codes for 61 comorbidities in our study were identified using CPRD GOLD data and further refined after comparing with codes used by other researchers in our department and other sources (8,9). Most of the code groups for comorbidities listed have been externally validated (10,11). A final list of codes was shared with our general practitioner collaborator for input and verification. Finally, the amended codes were reviewed and agreed by the research team. A summary of the disease list with primary Read codes is given in Supplementary Table S1.1.

The conditions were identified in the database using Read codes. We did not include musculoskeletal conditions as an outcome because of the possible bias towards prescriptions of studied analgesics, but musculoskeletal conditions were used for propensity score estimation.

# Study methods

Analyses were carried out using Stata and R. We analysed exposures for the different analgesics separately. A propensity score adjusted time varying exposure (analgesics) analysis was undertaken using a multivariable Cox model to estimate adjusted hazard ratios (HR) and 95% confidence intervals (CI) for each of the nine comorbidity outcomes (12). In the main analysis participants were followed up from the index date until the earliest of the outcome diagnosis, death, five years after the index date or the end of the study (31st Dec 2019).

Propensity scores (PS) for the prescription of each analgesic exposure were estimated separately using a logistic regression model including covariates such as age, sex, BMI, smoking, alcohol, musculoskeletal and all conditions at index date listed in Supplementary Table S1.2. The summary values of the scores in both exposed and non-exposed groups were evaluated. The purpose of using PS adjusted model instead of PS matching (a commonly used method) was to avoid a large reduction in study participant numbers and to prevent overmatching. We used the ‘Twang’ package in R to estimate the scores and evaluate the balances between exposed and non-exposed groups using ‘effect size’ and ‘Kolmogorov-Smirnov’ (KS) score. Details of the PS scores calculation and model performance are shown in Supplementary Figures S1.1, S1.2 and Table S1.3.

Participants were stratified into four strata according to initial prescription status with each analgesic (D) separately at exposure period: ‘No OA and no drugs’ (OA-D-), ‘OA and no drugs’ (OA+D-), ‘No OA and drugs’ (OA-D+), and ‘OA and drugs’ (OA+D+). An individual could belong to OA-D- group at the index date and move to OA-D+ group after the prescription date (Figure 1). Unadjusted and adjusted HRs and 95% CI were calculated for all groups against the reference group (OA-D-). Participants were grouped as unexposed until first prescription and exposed afterwards. The proportional hazards assumption was tested using the Schoenfeld test. In the adjusted model, the estimated propensity score was adjusted for. Cumulative hazards and incidence rates (per 1000 person-years) were reported for each comorbidity outcome. Furthermore, we assessed the HRs of incident comorbidities across different quintiles of average number of prescriptions for ‘dose-response’ estimations. The multiple testing problem was addressed using ‘False discovery rate’ ‘Benjamini- Hochberg’ approach, ‘FDR’ package in R.

Multiplicative interactions between OA and analgesic exposure for each incident comorbidity were estimated using the HR by including an interaction term (OA*Drug) in the final adjusted model. Additive interactions were calculated for outcomes with positive multiplicative interactions using three indicators proposed by Rothman et al (13). We reported relative excessive risk due to interaction (RERI), attributable proportion (AP) and synergy index (SI), each with respective 95% CI. Details of methods of calculation are given in Supplementary File 1 (Supplementary table S1.4). If the RERI and AP are equal to 0 and SI is equal to 1 that suggests absence of additive interactions. RERI > 0, AP>0, & SI>1 implies synergetic interaction, i.e., the combined action between two exposures in the additive model is greater than the sum of the individual effects. However, if RERI < 0, AP<0, & SI<1 it implies an antagonistic interaction, meaning that in the presence of two exposures in an additive model, the action of one exposure diminishes the effect of the other (13).

We also performed mediation analyses to explore the proportion of the association between OA and comorbidities mediated by analgesics in Stata using the ‘med4way’ package. Total natural direct effect (TNDE) and total natural indirect effect (TNIE) were estimated in adjusted model to explain the mediator role. TNIE represented the effect of OA on comorbidities in the presence of mediators (analgesics) and TNDE represented the effect without the mediators (i.e., no analgesics). The proportion of association by the mediator was reported to quantify the magnitude of the mediation (14).

# Table S1.1 List of the conditions and codes used in the study

| **Group** | **Diseases/ groups** | **Initials of major Read codes /BNF codes** |
| --- | --- | --- |
| Musculoskeletal | Rheumatoid Arthritis | N040.0; N040..; N043… ;N04y… |
|  | Back pain | N11….; N12…; N14….; N3…; Nyu…; S10.…; S49…; S57… |
|  | Gout | C34…; N02z…; N02y…; N022..; N021… |
|  | Osteoporosis | N33… |
|  | Fibromyalgia | N248.00; N239.00; F286… |
|  | Osteoarthritis | N05…; N051… |
| Cardio-vascular | Coronary Heart Disease (Myocardial infraction, Valvular disease, Ischemic heart disease, Angina) | G3…00; G30….; G30z….; G31…; G33…; G34…; G35…; G54….; G57….; Gyu… |
|  | Arterial/Venous (Atherosclerosis/aneurysm/ Embolism) | G70…; G71…; G72….; G74… |
|  | Heart failure (Congestive cardiac failure, heart failure) | G580…; G81….; G232.00; G234.00 |
|  | Hypertension | G20…; G21…; G24…; G25…; G26… |
|  | Peripheral Vascular Disease (Raynaud’s Disease, Buerger’s Disease, Intermittent Claudication) | G73… |
| Respiratory | Asthma | 663…; 66Y… |
|  | COPD | H31…; H32…; H33… |
| Renal | Chronic Kidney Disease (Stage III) | 1Z12.00; 1Z13.00; 1Z14.00; 1Z15.00; 1Z16.00; K01…; K02… |
|  | Renal stone | 4G4…; 7B07…; K120…. |
| Neurological | Stroke (Both Ischemic and Haemorrhagic) | 9Om0.00; 9Om…; 8HBJ.oo; G60…; G61…; F22…; G64…; G63… |
|  | Dementia | E00…; Eu0…; F11…. |
|  | Parkinson’s Disease | F12… |
|  | Migraine | F26…. |
| Psychiatric | Depression | Eu1…; Eu3… |
|  | Psychosis | Eu2Z.11; Eu0z.11 |
|  | Schizophrenia | Eu2… |
| Metabolic/Endocrine | High Cholesterol | C324.00; C322.00; C328.00 |
|  | Diabetes Mellitus | 9OL...00; 2BB…; 2G5…; 66A…; C10…; F42… |
|  | Hyperthyroid | C02… |
|  | Hypothyroidism | C03…; C04… |
| Gastrointestinal | Gastritis (Gastric Ulcer, Duodenal Ulcer without haemorrhage, gastritis) | J11…; J12…; J15…14C1… |
|  | Gastrointestinal bleed | J110…; J111….; J120…; J121…; J13….; J14…. J681… |
| Hepatic | Gall bladder stone | J65…; 4G2… |
| (Liver) | Liver Disease (Cirrhosis and Hepatitis)  Inflammatory Bowel Disease (Crohn’s Disease, Ulcerative colitis) | J61…; J63…; A70… |
|  | Irritable Bowel Syndrome | J521… |
| Cancer | Leukaemia |  |
|  | Solid cancer |  |
| NSAIDs | Oral NSAIDs | 10010100, 10010450, 4070100, 7010300, … |
| Opioids | Oral opioids | 4070200, 3090100, 1040200, 4105200, … |
| Paracetamol |  | 4070100 |

## Propensity score calculation

Propensity scores (PS) for the prescription of each analgesic exposure were estimated separately using a logistic regression model including covariates such as age, sex, BMI, smoking, alcohol, musculoskeletal and all conditions at index date listed in Supplementary Table S1.2. The summary values of the scores in both exposed and non-exposed groups were evaluated. The purpose of using PS adjusted model instead of PS matching (a commonly used method) was to avoid a large reduction in study participant numbers and to prevent overmatching. We used the ‘Twang’ package in R to estimate the scores and evaluate the balances between exposed and non-exposed groups using ‘effect size’ and ‘Kolmogorov-Smirnov’ (KS) score. Details of the PS scores calculation and model performance are shown in Supplementary Figures S1.1, S1.2 and Table S1.3.

We used logistic regression model to estimate propensity scores for prescription of NSAIDs, opioids and paracetamol using the covariates listed in Table S 1.2 .

Then, following steps were done to evaluate the PS score

Step 1: Estimate propensity score weights.

This was done in R. We calculated the weights using ‘inverse variance weight’ methods.

Step 2: Evaluate the quality of the propensity score weights in terms of balance – the balance was tested using ‘effect size’ and KS score. Lower is the KS score better is the balance.

# Table S1.2 Covariates included in propensity score estimation

| Alcohol status  Smoking status  Gender  Age group  BMI Group  **Medication at index date for respective group**  NSAIDs  Opioids  Paracetamol | **Conditions recorded at index date**  Musculoskeletal  Cardiovascular  Cancer  Endocrine  Gastrointestinal  Kidney / Renal  Liver  Neurological  Psychiatric  Respiratory |
| --- | --- |

# Figure S1.1 Estimated propensity scores for three types of analgesics

# Table S1.3 Comparison of model performance after weight calculation

|  | NSAIDs | | Opioids | | Paracetamol | |
| --- | --- | --- | --- | --- | --- | --- |
|  | Unweighted | Weighted | Unweighted | Weighted | Unweighted | Weighted |
| Max effect size | 0.49 | 0.06 | 0.51 | 0.04 | 0.58 | 0.03 |
| Mean effect size | 0.10 | 0.02 | 0.12 | 0.01 | 0.12 | 0.008 |
| Maximum KS | 0.22 | 0.02 | 0.23 | 0.06 | 0.25 | 0.08 |
| Mean KS | 0.04 | 0.007 | 0.05 | 0.01 | 0.05 | 0.009 |

KS- Komlogorov sminroff test

# Figure S1.2 Change in KS score before and after PS weighting

# Table S1.4 Notation and measures additive interaction calculation

| Measures | Reference value | Interpretation |
| --- | --- | --- |
| Relative excessive risk due to interaction (RERI) | 0 | =0 – no additive interaction  >0 – super-additive interaction  <0 – sub-additive interaction |
| Attributable proportion (AP) due to interaction | 0 | =0 - no proportion from the interaction  >0 – positive contribution from interaction  <0 – negative contribution from interaction |
| Rothman’s Synergy index (SI) | 1 | =1 – no additive interaction  >1 – super-additive interaction  <1 – sub-additive interaction |

Interaction is consistent if	RERI>0 & AP>0 & SI >0

or

RERI<0 & AP<0 & SI <0

|  | Paracetamol | | NSAIDs | | Opioids | |
| --- | --- | --- | --- | --- | --- | --- |
|  | No  N= 404452 | Yes  N=118094 | No  N=301194 | Yes  N=221352 | No  N=256461 | Yes  N=266085 |
| Osteoarthritis (n, %) | 184799 (45.69) | 76474 (64.76) | 115955 (38.50%) | 145318 (65.65%) | 95070 (37.07%) | 166203 (62.46%) |
| Mean age at index date (SD) | 58.19 (12.42) | 65.58 (12.70) | 60.03 (13.21) | 59.62 (12.36) | 57.99 (12.51) | 61.66 (12.94) |
| Mean age at index date in men (SD) | 58.49 (12.41) | 65.27 (11.84) | 59.69 (12.99) | 60.04 (12.00) | 58.20 (12.59) | 61.59 (12.36) |
| Mean age at index date in women (SD) | 57.95 (12.42) | 65.76 (13.18) | 60.29 (13.38) | 59.33 (12.60) | 57.82 (12.44) | 61.70 (13.31) |
| Mean BMI (kg/m^2^) (SD) | 27.32 (5.28) | 28.34 (5.88) | 26.93 (5.17) | 28.39 (5.67) |  |  |
| **Gender** |  |  |  |  |  |  |
| Men | 177013 (43.77) | 43815 (37.10) | 130242 (43.24) | 90586 (40.92) | 114410 (44.61) | 106418 (39.99) |
| Women | 227439 (56.23) | 74279 (62.90) | 170952 (56.76) | 130766 (59.08) | 142051 (55.39) | 159667 (60.01) |
| **BMI (kg/m^2^)** |  |  |  |  |  |  |
| <18.5 (Underweight) | 6160 (1.52) | 2649 (2.24) | 5900 (1.96) | 2909 (1.31) | 4084 (1.59) | 4725 (1.78) |
| 18.5- 24.9 (Normal) | 136595 (33.77) | 31732 (26.87) | 108135 (35.90) | 60192 (27.19) | 95613 (37.28) | 72714 (27.33) |
| 25.0-29.9 (Overweight) | 146717 (36.28) | 40131 (33.98) | 107578 (35.72) | 79270 (35.81) | 94476 (36.84) | 92372 (34.72) |
| ≥30 (Obese) | 101702 (25.15) | 38667 (32.74) | 68660 (22.80) | 71709 (32.40) | 54046 (21.07) | 86323 (32.44) |
| Missing | 13278 (3.28) | 4915 (4.16) | 10921 (3.63) | 7272 (3.29) | 8242 (3.21) | 9951 (3.74) |
| **Alcohol consumption (units/week)** |  |  |  |  |  |  |
| Never | 64152 (15.86) | 29528 (25.00) | 52127 (17.31) | 41553 (18.77) | 37470 (14.61) | 56210 (21.12) |
| Ex-drinker | 8318 (2.06) | 4449 (3.77) | 6883 (2.29) | 5884 (2.66) | 4253 (1.66) | 8514 (3.20) |
| Current 1-9 | 140146 (34.65) | 36567 (30.96) | 102727 (34.11) | 73986 (33.42) | 91275 (35.59) | 85438 (32.11) |
| Current >=10 | 82879 (20.49) | 16000 (13.55) | 57930 (19.23) | 40949 (18.50) | 54957 (21.90) | 43922 (16.51) |
| Current Unknown | 90164 (22.29) | 26398 (22.35) | 66394 (22.04) | 50168 (22.66) | 56154 (21.90) | 60408 (22.70) |
| Missing | 18793 (4.65) | 5152 (4.36) | 15133 (5.02) | 8812 (3.98) | 12352 (4.82) | 11593 (4.36) |
| **Smoking Status** |  |  |  |  |  |  |
| Never smoked | 222730 (55.07) | 59337 (50.25) | 167887 (55.74) | 114180 (51.58) | 150314 (58.61) | 131753 (49.52) |
| Ex-smoker | 103849 (25.68) | 36557 (30.96) | 79013 (26.23) | 61393 (27.74) | 62734 (24.46) | 77672 (29.19) |
| Current smoker | 77041 (19.05) | 21972 (18.61) | 53606 (17.80) | 45407 (20.51) | 42802 (16.69) | 56211 (21.13) |
| Missing | 832 (0.21) | 228 (0.19) | 688 (0.23) | 372 (0.17) | 611 (0.24) | 449 (0.17) |
| **Analgesics prescription ever** |  |  |  |  |  |  |
| Paracetamol | NA | NA | 45764 (15.19) | 72330 (32.68) | 38 (0.01) | 118056 (44.37) |
| NSAIDs | 149022 (36.85) | 72330 (61.25) | NA | NA | 56395 (21.99) | 164957 (61.99) |
| Opioids | 148029 (36.60) | 118056 (99.97) | 101128 (33.58) | 164957 (74.52) | NA | NA |
| Proton pump inhibitors (PPI) | 194015 (47.97) | 78435 (66.42) | 73253 (24.32) | 80507 (36.37) | 64685 (25.22) | 89089 (33.48) |
| **Comorbidities at index date** |  |  |  |  |  |  |
| Cancer | 16972 (4.20) | 7717 (6.53) | 14240 (4.73) | 10449 (4.72) | 10019 (3.91) | 14670 (5.51) |
| Cardiovascular | 125031 (30.91) | 58028 (49.14) | 102068 (33.89) | 80991 (36.59) | 72129 (28.12) | 110930 (41.69) |
| Endocrine | 85092 (21.04) | 35951 (30.44) | 65232 (21.66) | 55811 (25.21) | 48619 (18.69) | 72424 (27.22) |
| Gastrointestinal | 52763 (13.05) | 20353 (17.23) | 38609 (12.82) | 34507 (15.59) | 27886 (10.87) | 45230 (17.00) |
| Renal | 17715 (4.38) | 9217 (7.80) | 15367 (5.10) | 11565 (5.22) | 10085 (3.93) | 16947 (6.33) |
| Liver | 20512 (5.07) | 9810 (8.31) | 15431 (5.12) | 14891 (6.73) | 10414 (4.06) | 19908 (7.48) |
| Neurological | 60723 (15.01) | 23256 (19.69) | 45072 (14.96) | 38907 (17.58) | 34648 (13.51) | 49331 (18.54) |
| Psychological | 107946 (26.69) | 38591 (32.68) | 73374 (24.36) | 73163 (33.05) | 58058 (22.64) | 88479 (33.25) |
| Respiratory | 64542 (15.96) | 25142 (21.29) | 48186 (16.00) | 41498 (18.75) | 34942 (13.62) | 54742 (20.57) |

# Table S1.5 Descriptive statistics of the study population across exposure groups

NSAIDs- Non steroidal anti-inflammatory drugs; SD- Standard deviation; NA- Not applicable

# Table S1.6 Incidence rates of comorbidities per 1000 person-years over five years after the index date across different groups for three analgesics

|  | Incidence Rates per 1000 person-years (95% CI) | | | | | | | | |
| --- | --- | --- | --- | --- | --- | --- | --- | --- | --- |
|  | Cancer | Cardiovascular | Endocrine | Gastrointestinal | Renal | Liver | Neurological | Psychological | Respiratory |
| **Paracetamol** |  |  |  |  |  |  |  |  |  |
| no OA, no Drug | 6.13 (5.98-6.28) | 35.04 (34.60-35.49) | 25.69 (25.35-26.05) | 6.99 (6.83-7.17) | 10.86 (10.66-11.07) | 3.47 (3.36-3.59) | 11.24 (11.02-11.46) | 13.80 (13.54-14.06) | 7.69 (7.51-7.87) |
| OA, no drug | 7.90 (7.72-8.09) | 40.98 (40.46-41.51) | 30.19 (29.78-30.60) | 10.38 (10.15-10.60) | 11.37 (11.15-11.60) | 4.74 (4.60-4.89) | 13.25 (12.99-13.50) | 19.49 (19.15-19.83) | 9.67 (9.46-9.90) |
| No OA, drug | 10.89 (9.95-11.92) | 57.67 (54.13-61.44) | 36.80 (34.62-39.12) | 11.96 (10.91-13.11) | 33.01 (31.26-34.85) | 6.90 (6.16-7.73) | 24.22 (22.62-25.92) | 20.18 (18.65-21.84) | 14.42 (13.23-15.71) |
| OA and drug | 14.17 (13.46-14.91) | 64.48 (62.11- 66.94) | 41.42 (39.90-43.00) | 14.60 (13.83-15.41) | 29.03 (27.97-30.12) | 7.21 (6.71-7.75) | 22.41 (21.43-23.44) | 25.61 (24.44-26.83) | 14.17 (13.39-15.00) |
| **NSAIDs** |  |  |  |  |  |  |  |  |  |
| no OA, no Drug | 5.69 (5.53-5.84) | 35.09 (34.64-35.56) | 24.21 (23.86-24.57) | 6.19 (6.02-6.35) | 11.04 (10.83-11.25) | 3.22 (3.11-3.34) | 10.53 (10.31-10.75) | 12.19 (11.94-12.44) | 7.03 (6.86-7.21) |
| OA, no drug | 7.41 (7.21-7.61) | 38.48 (37.90-39.07) | 25.99 (25.56-26.42) | 8.58 (8.35-8.81) | 11.49 (11.25-11.74) | 4.18 (4.03-4.33) | 11.99 (11.72-12.27) | 15.67 (15.33-16.01) | 8.18 (7.96-8.42) |
| No OA, drug | 8.23 (7.59-8.91) | 48.97 (46.94-51.09) | 32.42 (30.93-33.98) | 11.02 (10.24-11.86) | 17.61 (16.67-18.62) | 4.99 (4.50-5.53) | 13.73 (12.84-14.69) | 18.41 (17.26-19.64) | 9.85 (9.10-10.66) |
| OA and drug | 9.36 (8.98-9.76) | 54.24 (53.03-55.47) | 37.14 (36.24-38.06) | 12.89 (12.41-13.78) | 16.42 (15.91-16.95) | 5.64 (5.34-5.95) | 14.91 (14.39-15.45) | 23.75 (23.01-24.52) | 11.45 (10.99-11.93) |
| **Opioids** |  |  |  |  |  |  |  |  |  |
| no OA, no Drug | 5.87 (5.72-6.03) | 34.16 (33.72-34.62) | 25.08 (24.73-25.44) | 6.59 (6.42-6.76) | 10.29 (10.08-10.49) | 3.20 (3.08-3.31) | 10.94 (10.72-11.17) | 13.18 (12.92-13.44) | 7.33 (7.15-7.52) |
| OA, no drug | 7.20 (7.01-7.40) | 38.00 (37.44-38.56) | 28.00 (27.57-28.45) | 9.39 (9.15-9.63) | 9.87 (9.64-10.11) | 4.11 (3.96-4.26) | 12.48 (12.21-12.77) | 17.57 (17.21-17.93) | 8.65 (8.42-8.88) |
| No OA, drug | 10.01 (9.45-10.60) | 51.26 (49.42-53.17) | 35.10 (33.82-36.43) | 12.28 (11.61-12.98) | 23.49 (22.60-24.41) | 6.95 (6.49-7.46) | 18.35 (17.51-19.23) | 22.06 (21.03-23.13) | 13.23 (12.52-13.98) |
| OA and drug | 11.72 (11.34-12.12) | 57.02 (55.79-58.27) | 39.66 (38.79-40.55) | 14.32 (13.85-14.80) | 21.14 (20.60-21.68) | 7.13 (6.83-7.45) | 18.29 (17.76-18.84) | 26.90 (26.16-27.66) | 13.85 (13.83-14.34) |

CI- Confidence interval; IR- Incident rate; OA- Osteoarthritis; D- Drug

# Figure S1.3. Dose-response association of analgesics in OA, non-OA, and total

a. Paracetamol

b. NSAIDs

c. Opioids

Expanded version of the figure is available in supplementary Table S2.5 to S2.7.

aHR- Adjusted hazard ratio. Adjusted for age at index date, sex, smoking, alcohol, propensity score, other comorbidities at index date and other analgesics

Psych-Psychological; Neuro- Neurological; CVD- Cardiovascular; GI- Gastrointestinal; Endo- Endocrine; Resp- Respiratory

This was estimated by first calculating the total number of prescriptions made between the index date and the date of the end of follow-up or death/left practice if earlier, and then dividing the total number of prescriptions by the number of follow-up years.

The risk sharply increased from Q4 to Q5 in all the groups for all the comorbidities. These increases in risk were more prominent in the OA compared to the non-OA group.

Reference

1.	Quality and Outcome Framework (QOF) [Internet]. [cited 2018 Mar 21]. Available from: https://digital.nhs.uk/article/8910/Quality-and-Outcome-Framework-QOF-Indicators-No-Longer-In-QOF-INLIQ-Enhanced-Services-ES-Vaccinations-and-Immunisations-V-I-and-GMS-Core-Contract-CC-extraction-specifications-business-rules-

2.	Medicare C for, Baltimore MS 7500 SB, Usa M. CC_Main [Internet]. 2017 [cited 2018 Oct 24]. Available from: https://www.cms.gov/Research-Statistics-Data-and-Systems/Statistics-Trends-and-Reports/Chronic-Conditions/CC_Main.html

3.	Charlson ME, Pompei P, Ales KL, MacKenzie CR. A new method of classifying prognostic comorbidity in longitudinal studies: Development and validation. J Chronic Dis. 1987 Jan 1;40(5):373–83.

4.	Swain S, Sarmanova A, Coupland C, Doherty M, Zhang W. Comorbidities in Osteoarthritis: A systematic review and meta-analysis of observational studies. 2019 June 17 [cited 2019 June 30]; Available from: http://doi.wiley.com/10.1002/acr.24008

5.	Sarmanova A, Fernandes GS, Richardson H, Valdes AM, Walsh DA, Zhang W, et al. Contribution of central and peripheral risk factors to prevalence, incidence and progression of knee pain: a community-based cohort study. Osteoarthritis Cartilage. 2018 Nov;26(11):1461–73.

6.	Charlson M, Szatrowski TP, Peterson J, Gold J. Validation of a combined comorbidity index. J Clin Epidemiol. 1994 Nov 1;47(11):1245–51.

7.	Quan H, Li B, Couris CM, Fushimi K, Graham P, Hider P, et al. Updating and Validating the Charlson Comorbidity Index and Score for Risk Adjustment in Hospital Discharge Abstracts Using Data From 6 Countries. Am J Epidemiol. 2011 Mar 15;173(6):676–82.

8.	Springate DA, Kontopantelis E, Ashcroft DM, Olier I, Parisi R, Chamapiwa E, et al. ClinicalCodes: An Online Clinical Codes Repository to Improve the Validity and Reproducibility of Research Using Electronic Medical Records. Petersen I, editor. PLoS ONE. 2014 June 18;9(6):e99825.

9.	Payne RA, Mendonca SC, Elliott MN, Saunders CL, Edwards DA, Marshall M, et al. Development and validation of the Cambridge Multimorbidity Score. Can Med Assoc J. 2020 Feb 3;192(5):E107–14.

10.	Deyo R. Adapting a clinical comorbidity index for use with ICD-9-CM administrative databases. J Clin Epidemiol. 1992 June;45(6):613–9.

11.	Khan NF, Harrison SE, Rose PW. Validity of diagnostic coding within the General Practice Research Database: a systematic review. Br J Gen Pract. 2010 Mar 1;60(572):e128–36.

12.	Desai RJ, Franklin JM. Alternative approaches for confounding adjustment in observational studies using weighting based on the propensity score: a primer for practitioners. BMJ. 2019 Oct 23;l5657.

13.	Rothman KJ. Epidemiology: an introduction. 2nd ed. New York, NY: Oxford University Press; 2012. 268 p.

14.	Discacciati A, Bellavia A, Lee JJ, Mazumdar M, Valeri L. Med4way: a Stata command to investigate mediating and interactive mechanisms using the four-way effect decomposition. Int J Epidemiol. 2019 Feb 1;48(1):15–20.
