# Supplementary material for: Association of analgesics prescription with incident comorbidities in people with osteoarthritis—a UK primary care database cohort study
Source: Rheumatol Adv Pract. 2026 Aug 7;10(3):rkag098. doi: 10.1093/rap/rkag098 (PMC13529369; doi:10.1093/rap/rkag098)
Supplement: Supplementary file 1 [file rkag098_supplementary_data.zip › Analgesic_Supplementary File2.docx]

Supplementary file 2

Contents

[Table S2.1 Frequency of proton pump inhibitors (PPI) prescriptions across three analgesic types	1](#_Toc125924579)

Table S2.2. Risk of developing comorbidities in one, five, ten and 22 years among OA and paracetamol group…….2

Table S2.3 Risk of developing comorbidities in one, five, ten and 22 years among OA and NSAIDs group………………3

Table S2.4 Risk of developing comorbidities in one, five, ten and 22 years among OA and opioids group……………..4

[Table S2.5 Risk of comorbidities in analgesics (adjusted for PPI)	5](#_Toc125924580)

[Table S2.6 Dose-response association of paracetamol use with comorbidities	6](#_Toc125924581)

[Table S2.7 Dose-response association of NSAIDs use with comorbidities	7](#_Toc125924582)

[Table S2.8 Dose-response association of opioids use with comorbidities	8](#_Toc125924583)

[Figure S2.1 Cumulative hazard of risk of comorbidities among paracetamol users	9](#_Toc125924584)

[Figure S2.2 Cumulative hazard of risk of comorbidities among NSAIDs users	10](#_Toc125924585)

[Figure S2.3 Cumulative hazard of risk of comorbidities among opioids users	11](#_Toc125924586)

[Table S2.9 Classification of NSAIDs	12](#_Toc125924587)

[Table S2.10 Types of NSAIDs and the risk of comorbidities	13](#_Toc125924588)

# Table S2.1 Frequency of proton pump inhibitor (PPI) prescriptions across the three analgesic types

|  | Paracetamol  (N=118094)  N (%) | NSAIDs  (N=228161)  N (%) | Opioids  (N=266085)  N (%) |
| --- | --- | --- | --- |
| PPI (No) | 26367 (22.33) | 61712 (27.05) | 68963 (25.92) |
| PPI (Yes) | 91727 (77.67) | 166449 (72.95) | 197122 (74.08) |

Table S2.2. Risk of developing comorbidities in one, five, ten and 22 years after index date among OA and paracetamol groups

|  |  |  | One year | | 5 years | | 10 years | | 22 years | |
| --- | --- | --- | --- | --- | --- | --- | --- | --- | --- | --- |
|  | OA | Drug | uHR(95% CI) | aHR(95% CI)^#^ | uHR(95% CI) | aHR(95% CI)^#^ | uHR(95% CI) | aHR(95% CI)^#^ | uHR(95% CI) | aHR(95% CI)^#^ |
| Cancer | - | - | 1 | 1 | 1 | 1 | 1 | 1 | 1 | 1 |
|  | + | - | **1.39 (1.29-1.49)** | **1.17 (1.08-1.26)** | **1.29 (1.25-1.34)** | **1.09 (1.06-1.13)** | **1.25 (1.22-1.29)** | **1.07 (1.04-1.09)** | **1.23 (1.19-1.26)** | **1.03 (1.01-1.06)** |
|  | - | + | **2.05 (1.46-2.87)** | 1.11 (0.79-1.56) | **1.72 (1.56-1.89)** | 0.93 (0.84-1.02) | **1.61 (1.52-1.71)** | 0.87 (0.82-0.93) | **1.83 (1.74-1.92)** | 0.98 (0.93-1.03) |
|  | + | + | **3.71 (3.16-4.36)** | **2.15 (1.82-2.53)** | **2.24 (2.12-2.38)** | **1.30 (1.22-1.38)** | **2.00 (1.93-2.09)** | **1.16 (1.11-1.21)** | **2.13 (2.05-2.20)** | **1.22 (1.18-1.27)** |
| Cardiovascular | - | - | 1 | 1 | 1 | 1 | 1 | 1 | 1 | 1 |
|  | + | - | **1.26 (1.22-1.31)** | 1.10 (1.06-1.14) | **1.16 (1.14-1.19)** | 1.02 (1.00-1.04) | **1.13 (1.11-1.15)** | 1.00 (0.99-1.01) | **1.12 (1.10-1.14)** | 0.99 (0.98-1.01) |
|  | - | + | **1.82 (1.46-2.27)** | 1.06 (0.85-1.31) | **1.66 (1.55-1.77)** | 0.98 (0.92-1.05) | **1.51 (1.45-1.59)** | 0.91 (0.87-0.96) | **1.49 (1.44-1.55)** | 0.92 (0.88-0.95) |
|  | + | + | **2.59 (2.31-2.89)** | **1.59 (1.42-1.79)** | **1.86 (1.78-1.93)** | **1.17 (1.12-1.22)** | **1.71 (1.66-1.76)** | **1.09 (1.06-1.13)** | **1.66 (1.61-1.70)** | **1.08 (1.05-1.10)** |
| Endocrine | - | - | 1 | 1 | 1 | 1 | 1 | 1 | 1 | 1 |
|  | + | - | **1.18 (1.13-1.22)** | **1.07 (1.03-1.11)** | **1.17 (1.15-1.19)** | **1.06 (1.04-1.08)** | **1.16 (1.14-1.18)** | **1.05 (1.04-1.07)** | **1.16 (1.14-1.17)** | **1.05 (1.04-1.07)** |
|  | - | + | **1.92 (1.57-2.34)** | **1.31 (1.07-1.60)** | **1.43 (1.34-1.52)** | 0.96 (0.89-1.02) | **1.33 (1.28-1.39)** | 0.91 (0.87-0.95) | **1.29 (1.25-1.34)** | 0.89 (0.86-0.93) |
|  | + | + | **2.13 (1.89-2.39)** | **1.51 (1.34-1.71)** | **1.61 (1.54-1.67)** | **1.12 (1.08-1.17)** | **1.53 (1.48-1.57)** | **1.09 (1.05-1.12)** | **1.46 (1.42-1.50)** | **1.05 (1.02-1.08)** |
| Gastrointestinal | - | - | 1 | 1 | 1 | 1 | 1 | 1 | 1 | 1 |
|  | + | - | **1.47 (1.38-1.57)** | **1.33 (1.24-1.42)** | **1.48 (1.44-1.53)** | **1.34 (1.30-1.39)** | **1.46 (1.42-1.50)** | **1.33 (1.29-1.36)** | **1.45 (1.41-1.48)** | **1.31 (1.28-1.35)** |
|  | - | + | **2.04 (1.51-2.77)** | **1.42 (1.04-1.94)** | **1.74 (1.58-1.92)** | **1.21 (1.10-1.33)** | **1.65 (1.55-1.76)** | **1.14 (1.06-1.22)** | **1.60 (1.51-1.69)** | **1.12 (1.05-1.18)** |
|  | + | + | **2.49 (2.09-2.96)** | **1.80 (1.50-2.15)** | **2.11 (1.99-2.24)** | **1.53 (1.43-1.63)** | **2.05 (1.96-2.14)** | **1.48 (1.41-1.54)** | **2.04 (1.97-2.12)** | **1.48 (1.42-1.54)** |
| Liver | - | - | 1 | 1 | 1 | 1 | 1 | 1 | 1 | 1 |
|  | + | - | **1.37 (1.25-1.51)** | **1.23 (1.12-1.35)** | **1.37 (1.30-1.43)** | **1.22 (1.17-1.28)** | **1.37 (1.32-1.42)** | **1.23 (1.18-1.27)** | **1.36 (1.31-1.40)** | **1.21 (1.17-1.26)** |
|  | - | + | **2.61 (1.82-3.77)** | **1.75 (1.21-2.53)** | **1.96 (1.74-2.20)** | **1.28 (1.13-1.45)** | **1.78 (1.63-1.93)** | **1.16 (1.07-1.27)** | **1.71 (1.60-1.83)** | **1.13 (1.05-1.21)** |
|  | + | + | **2.45 (1.93-3.11)** | **1.71 (1.34-2.18)** | **2.05 (1.89-2.22)** | **1.40 (1.29-1.53)** | **2.07 (1.96-2.19)** | **1.42 (1.33-1.51)** | **2.01 (1.91-2.10)** | **1.38 (1.31-1.46)** |
| Neurological | - | - | 1 | 1 | 1 | 1 | 1 | 1 | 1 | 1 |
|  | + | - | **1.12 (1.06-1.19)** | 0.98 (0.93-1.04) | **1.18 (1.15-1.21)** | 1.02 (0.99-1.04) | **1.15 (1.13-1.18)** | 0.99 (0.97-1.02) | **1.12 (1.10-1.15)** | 0.97 (0.95-0.99) |
|  | - | + | **2.31 (1.82-2.94)** | **1.43 (1.12-1.82)** | **2.15 (2.01-2.31)** | **1.27 (1.18-1.36)** | **2.14 (2.04-2.24)** | **1.24 (1.18-1.30)** | **2.13 (2.05-2.21)** | **1.22 (1.17-1.27)** |
|  | + | + | **2.31 (1.99-2.67)** | **1.49 (1.28-1.74)** | **1.99 (1.90-2.09)** | **1.24 (1.18-1.3)** | **1.95 (1.88-2.02)** | **1.19 (1.15-1.23)** | **1.88 (1.82-1.94)** | **1.13 (1.10-1.17)** |
| Psychological | - | - | 1 | 1 | 1 | 1 | 1 | 1 | 1 | 1 |
|  | + | - | **1.49 (1.42-1.56)** | **1.39 (1.32-1.47)** | **1.40 (1.37-1.44)** | **1.31 (1.28-1.35)** | **1.38 (1.35-1.41)** | **1.29 (1.27-1.32)** | **1.38 (1.35-1.41)** | **1.29 (1.26-1.32)** |
|  | - | + | **2.31 (1.85-2.90)** | **1.80 (1.43-2.26)** | **1.53 (1.41-1.66)** | **1.18 (1.08-1.28)** | **1.58 (1.49-1.67)** | **1.22 (1.15-1.29)** | **1.58 (1.50-1.66)** | **1.21 (1.16-1.28)** |
|  | + | + | **2.32 (2.01-2.67)** | **1.84 (1.53-2.13)** | **1.93 (1.83-2.03)** | **1.52 (1.44-1.6)** | **1.86 (1.79-1.94)** | **1.47 (1.41-1.54)** | **1.82 (1.76-1.88)** | **1.43 (1.38-1.49)** |
| Renal | - | - | 1 | 1 | 1 | 1 | 1 | 1 | 1 | 1 |
|  | + | - | **1.10 (1.04-1.17)** | 0.99 (0.83-0.94) | **1.06 (1.02-1.08)** | **0.84 (0.82-0.87)** | **1.06 (1.04-1.08)** | **0.85 (0.83-0.87)** | **1.04 (1.02-1.06)** | **0.84 (0.82-0.86)** |
|  | - | + | **3.08 (2.46-3.85)** | **1.43 (1.14-1.80)** | **2.88 (2.71-3.05)** | **1.30 (1.23-1.4)** | **2.60 (2.50-2.70)** | **1.17 (1.13-1.22)** | **2.46 (2.38-2.54)** | **1.13 (1.09-1.17)** |
|  | + | + | **3.57 (3.13-4.07)** | **1.81 (1.58-2.06)** | **2.55 (2.44-2.66)** | **1.27 (1.21-1.32)** | **2.29 (2.22-2.35)** | **1.14 (1.10-1.1)** | **2.17 (2.12-2.23)** | **1.09 (1.07-1.12)** |
| Respiratory | - | - | 1 | 1 | 1 | 1 | 1 | 1 | 1 | 1 |
|  | + | - | **1.18 (1.10-1.26)** | 1.05 (0.98-1.12) | **1.26 (1.21-1.29)** | **1.11 (1.08-1.16)** | **1.25 (1.21-1.28)** | **1.11 (1.09-1.15)** | **1.25 (1.22-1.28)** | **1.11 (1.09-1.14)** |
|  | - | + | **2.39 (1.81-3.15)** | **1.53 (1.15-2.02)** | **1.89 (1.74-2.07)** | **1.21 (1.1-1.32)** | **1.74 (1.63-1.85)** | **1.12 (1.05-1.19)** | **1.67 (1.58-1.75)** | **1.08 (1.03-1.14)** |
|  | + | + | **2.03 (1.68-2.45)** | **1.36 (1.12-1.65)** | **1.86 (1.75-1.98)** | **1.24 (1.16-1.32)** | **1.82 (1.74-1.90)** | **1.23 (1.17-1.29)** | **1.76 (1.69-1.82)** | **1.19 (1.14-1.24)** |

aHR- Adjusted hazard ratio; uHR-unadjusted hazard ratio

\# adjusted for age at index date, sex, smoking, alcohol, propensity score, other comorbidities at index date, and other analgesics

CI- Confidence interval, HR- Hazard ratio, NSAIDs- Non-steroidal anti-inflammatory drugs; OA-Osteoarthritis.

Bold indicates significant p value <0.05 after adjusting for multiple testing problem.

Table S2.3 Risk of developing comorbidities in one, five, ten and 22 years after index date among OA and NSAID groups

|  |  |  | One year | | 5 years | | 10 years | | 22 years | |
| --- | --- | --- | --- | --- | --- | --- | --- | --- | --- | --- |
|  | OA | Drug | uHR(95% CI) | aHR(95% CI^)#^ | uHR(95% CI) | aHR(95% CI)^)#^ | uHR(95% CI) | aHR(95% CI)^)#^ | uHR(95% CI) | aHR(95% CI)^)#^ |
| Cancer | - | - | 1 | 1 | 1 | 1 | 1 | 1 | 1 | 1 |
|  | + | - | 1.40 (1.29-1.51) | 1.34 (1.23-1.46) | 1.31 (1.26-1.36) | 1.25(1.2-1.3) | 1.28 (1.24-1.32) | 1.20 (1.17-1.24) | 1.24 (1.23-1.30) | 1.19 (1.16-1.23) |
|  | - | + | 1.59 (1.16-2.18) | 1.48 (1.08-2.03) | 1.38 (1.27-1.50) | 1.26(1.15-1.37) | 1.28 (1.21-1.35) | 1.15 (1.09-1.21) | 1.39 (1.33-1.46) | 1.26 (1.20-1.32) |
|  | + | + | 1.96 (1.71-2.26) | 1.82 (1.57-2.10) | 1.59 (1.51-1.67) | 1.42(1.35-1.5) | 1.51 (1.45-1.56) | 1.31 (1.26-1.36) | 1.53 (1.48-1.58) | 1.34 (1.29-1.39) |
| Cardiovascular | - | - | 1 | 1 | 1 | 1 | 1 | 1 | 1 | 1 |
|  | + | - | 1.15 (1.11-1.19) | 1.06 (1.02-1.10) | 1.09 (1.07-1.11) | 1.01(0.99-1.03) | 1.06 (1.05-1.08) | 0.98 (0.97-1.00) | 1.06 (1.04-1.08) | 0.99 (0.97-1.00) |
|  | - | + | 1.73 (1.50-2.00) | 1.47 (1.27-1.69) | 1.42 (1.35-1.48) | 1.21(1.16-1.27) | 1.31 (1.27-1.36) | 1.12 (1.09-1.16) | 1.28 (1.25-1.32) | 1.10 (1.07-1.13) |
|  | + | + | 2.10 (1.97-2.24) | 1.75 (1.64-1.88) | 1.56 (1.52-1.60) | 1.29(1.26-1.33) | 1.46 (1.43-1.49) | 1.20 (1.18-1.23) | 1.43 (1.40-1.46) | 1.18 (1.15-1.20) |
| Endocrine | - | - | 1 | 1 | 1 | 1 | 1 | 1 | 1 | 1 |
|  | + | - | 1.04 (0.99-1.08) | 0.98 (0.94-1.03( | 1.07 (1.05-1.09) | 1(0.98-1.03) | 1.07 (1.05-1.09) | 0.99 (0.98-1.01) | 1.07 (1.06-1.09) | 1.00 (0.98-1.01) |
|  | - | + | 1.65 (1.40-1.94) | 1.49 (1.26-1.75) | 1.33 (1.26-1.39) | 1.17(1.11-1.23) | 1.29 (1.25-1.33) | 1.13 (1.10-1.18) | 1.28 (1.25-1.32) | 1.13 (1.09-1.16) |
|  | + | + | 1.92 (1.78-2.07) | 1.71 (1.58-1.85) | 1.53 (1.48-1.57) | 1.31(1.27-1.35) | 1.47 (1.43-1.50) | 1.24 (1.22-1.28) | 1.44 (1.41-1.47) | 1.21 (1.19-1.24) |
| Gastrointestinal | - | - | 1 | 1 | 1 | 1 | 1 | 1 | 1 | 1 |
|  | + | - | 1.32 (1.22-1.41) | 1.19 (1.10-1.28) | 1.38 (1.33-1.43) | 1.26(1.21-1.31) | 1.36 (1.32-1.41) | 1.25 (1.21-1.29) | 1.36 (1.32-1.40) | 1.23 (1.19-1.27) |
|  | - | + | 1.39 (1.03-1.89) | 1.16 (0.86-1.56) | 1.81 (1.67-1.96) | 1.54(1.42-1.67) | 1.61 (1.52-1.70) | 1.32 (1.24-1.39) | 1.57 (1.50-1.65) | 1.33 (1.27-1.39) |
|  | + | + | 2.39 (2.11-2.68) | 1.94 (1.72-2.20) | 2.10 (2.00-2.20) | 1.73(1.64-1.82) | 2.02 (1.95-2.10) | 1.58 (1.51-1.64) | 1.99 (1.93-2.06) | 1.60 (1.55-1.66) |
| Liver | - | - | 1 | 1 | 1 | 1 | 1 | 1 | 1 | 1 |
|  | + | - | 1.29 (1.17-1.43) | 1.12 (1.01-1.25) | 1.30 (1.24-1.37) | 1.12(1.08-1.18) | 1.32 (1.26-1.37) | 1.14 (1.09-1.19) | 1.32 (1.27-1.37) | 1.14 (1.10-1.19) |
|  | - | + | 1.78 (1.24-2.58) | 1.39 (0.96-2.00) | 1.52 (1.36-1.69) | 1.18(1.06-1.32) | 1.48 (1.37-1.59) | 1.16 (1.08-1.25) | 1.40 (1.32-1.49) | 1.10 (1.04-1.17) |
|  | + | + | 1.74 (1.45-2.09) | 1.31 (1.09-1.59) | 1.72 (1.61-1.84) | 1.28(1.19-1.37) | 1.78 (1.69-1.86) | 1.30 (1.24-1.38) | 1.72 (1.64-1.79) | 1.25 (1.19-1.30) |
| Neurological | - | - | 1 | 1 | 1 | 1 | 1 | 1 | 1 | 1 |
|  | + | - | 1.03 (0.97-1.09) | 0.99 (0.93-1.06) | 1.14 (1.10-1.18) | 1.08(1.04-1.11) | 1.14 (1.11-1.17) | 1.08 (1.05-1.10) | 1.13 (1.11-1.16) | 1.06 (1.04-1.09) |
|  | - | + | 1.10 (0.83-1.45) | 1.03 (0.78-1.36) | 1.28 (1.19-1.38) | 1.16(1.08-1.25) | 1.23 (1.17-1.29) | 1.11 (1.06-1.16) | 1.18 (1.14-1.22) | 1.06 (1.02-1.09) |
|  | + | + | 1.52 (1.35-1.70) | 1.42 (1.26-1.59) | 1.40 (1.34-1.46) | 1.24(1.19-1.3) | 1.33 (1.28-1.36) | 1.16 (1.12-1.20) | 1.27 (1.24-1.30) | 1.09 (1.07-1.13) |
| Psychological | - | - | 1 | 1 | 1 | 1 | 1 | 1 | 1 | 1 |
|  | + | - | 1.28 (1.20-1.35) | 1.13 (1.06-1.19) | 1.27 (1.23-1.31) | 1.11(1.08-1.14) | 1.26 (1.23-1.29) | 1.10 (1.07-1.13) | 1.27 (1.24-1.30) | 1.10 (1.08-1.13) |
|  | - | + | 1.77 (1.42-2.21) | 1.42 (1.13-1.77) | 1.57 (1.46-1.68) | 1.24(1.16-1.33) | 1.52 (1.45-1.59) | 1.20 (1.14-1.26) | 1.48 (1.42-1.54) | 1.17 (1.11-1.21) |
|  | + | + | 2.63 (2.40-2.89) | 2.05 (1.87-2.26) | 1.99 (1.92-2.07) | 1.49(1.44-1.56) | 1.88 (1.83-1.94) | 1.39 (1.35-1.44) | 1.83 (1.78-1.88) | 1.34 (1.30-1.38) |
| Renal | - | - | 1 | 1 | 1 | 1 | 1 | 1 | 1 | 1 |
|  | + | - | 1.06 (1.00-1.13) | 0.98 (0.93-1.06) | 1.06 (1.03-1.09) | 0.98(0.95-1) | 1.05 (1.04-1.08) | 0.98 (0.95-1.00) | 1.05 (1.03-1.07) | 0.97 (0.95-0.99) |
|  | - | + | 2.07 (1.68-2.55) | 1.81 (1.47-2.23) | 1.48 (1.40-1.57) | 1.28(1.21-1.36) | 1.34 (1.29-1.39) | 1.16 (1.12-1.21) | 1.32 (1.28-1.36) | 1.14 (1.10-1.19) |
|  | + | + | 1.60 (1.43-1.80) | 1.38 (1.22-1.56) | 1.41 (1.36-1.47) | 1.18(1.13-1.23) | 1.39 (1.36-1.43) | 1.17 (1.13-1.20) | 1.37 (1.33-1.40) | 1.14 (1.11-1.17) |
| Respiratory | - | - | 1 | 1 | 1 | 1 | 1 | 1 | 1 | 1 |
|  | + | - | 1.02 (0.95-1.10) | 0.95 (0.88-1.02) | 1.16 (1.12-1.20) | 1.06(1.02-1.1) | 1.17 (1.13-1.21) | 1.06 (1.03-1.10) | 1.17 (1.14-1.21) | 1.06 (1.04-1.09) |
|  | - | + | 1.43 (1.07-1.91) | 1.24 (0.93-1.67) | 1.40 (1.29-1.52) | 1.19(1.09-1.3) | 1.39 (1.31-1.47) | 1.17 (1.11-1.24) | 1.35 (1.29-1.41) | 1.14 (1.09-1.19) |
|  | + | + | 1.85 (1.63-2.10) | 1.59 (1.39-1.81) | 1.62 (1.54-1.70) | 1.33(1.27-1.41) | 1.60 (1.54-1.65) | 1.29 (1.24-1.34) | 1.56 (1.51-1.61) | 1.24 (1.20-1.29) |

aHR- Adjusted hazard ratio; uHR-unadjusted hazard ratio

\# adjusted for age at index date, sex, smoking, alcohol, propensity score, other comorbidities at index date, and other analgesics

CI- Confidence interval, HR- Hazard ratio, NSAIDs- Non-steroidal anti-inflammatory drugs; OA-Osteoarthritis.

Bold indicates significant p value <0.05 after adjusting for multiple testing problem.

Table S2.4 Risk of developing comorbidities in one, five, ten and 22 years after index date among OA and opioid groups

|  |  |  | One year | | 5 years | | 10 years | | 22 years | |
| --- | --- | --- | --- | --- | --- | --- | --- | --- | --- | --- |
|  | OA | Drug | uHR(95% CI) | aHR(95% CI) | uHR(95% CI) | aHR(95% CI) | uHR(95% CI) | aHR(95% CI) | uHR(95% CI) | aHR(95% CI) |
| Cancer | - | - | 1 | 1 | 1 | 1 | 1 | 1 | 1 | 1 |
|  | + | - | 1.28 (1.18-1.38) | 1.12 (1.04-1.22) | 1.24 (1.19-1.28) | 1.1(1.06-1.15) | 1.20 (1.16-1.23) | 1.07 (1.04-1.10) | 1.16 (1.13-1.19) | 1.04 (1.01-1.07) |
|  | - | + | 1.96 (1.56-2.42) | 1.44 (1.16-1.79) | 1.66 (1.55-1.76) | 1.22(1.14-1.31) | 1.56 (1.49-1.63) | 1.13 (1.08-1.18) | 1.49 (1.44-1.54) | 1.07 (1.03-1.11) |
|  | + | + | 2.99 (2.67-3.36) | 2.18 (1.93-2.47) | 1.95 (1.87-2.04) | 1.4(1.33-1.47) | 1.80 (1.75-1.87) | 1.27 (1.22-1.31) | 1.69 (1.65-1.74) | 1.17 (1.13-1.21) |
| Cardiovascular | - | - | 1 | 1 | 1 | 1 | 1 | 1 | 1 | 1 |
|  | + | - | 1.20 (1.16-1.25) | 1.09 (1.05-1.13) | 1.10 (1.08-1.12) | 1.01(0.99-1.03) | 1.07 (1.05-1.09) | 0.98 (0.96-0.99) | 1.06 (1.04-1.08) | 0.97 (0.96-0.99) |
|  | - | + | 1.77 (1.57-2.01) | 1.38 (1.21-1.57) | 1.52 (1.47-1.58) | 1.19(1.14-1.24) | 1.41 (1.37-1.45) | 1.10 (1.07-1.13) | 1.38 (1.34-1.41) | **1.07 (1.05-1.10)** |
|  | + | + | 2.26 (2.11-2.42) | 1.73 (1.62-1.86) | 1.69 (1.65-1.73) | 1.28(1.24-1.32) | 1.58 (1.55-1.62) | 1.20 (1.17-1.22) | 1.54 (1.51-1.57) | **1.16 (1.13-1.19)** |
| Endocrine | - | - | 1 | 1 | 1 | 1 | 1 | 1 | 1 | 1 |
|  | + | - | 1.13 (1.09-1.18) | 1.03 (0.99-1.07) | 1.11 (1.09-1.13) | 1.02(1.01-1.04) | 1.09 (1.08-1.1) | 1.01 (0.99-1.03) | 1.09 (1.08-1.11) | 1.01 (1.00-1.03) |
|  | - | + | 1.74 (1.53-1.97) | 1.36 (1.20-1.55) | 1.40 (1.34-1.46) | 1.1(1.06-1.15) | 1.33 (1.29-1.37) | 1.06 (1.02-1.09) | 1.31 (1.28-1.34) | 1.04 (1.02-1.07) |
|  | + | + | 1.93 (1.79-2.07) | 1.50 (1.39-1.62) | 1.58 (1.54-1.62) | 1.22(1.18-1.26) | 1.51 (1.48-1.54) | 1.17 (1.14-1.19) | 1.48 (1.45-1.50) | 1.15 (1.12-1.17) |
| Gastrointestinal | - | - | 1 | 1 | 1 | 1 | 1 | 1 | 1 | 1 |
|  | + | - | 1.39 (1.30-1.49) | 1.21 (1.13-1.29) | 1.41 (1.36-1.46) | 1.24(1.2-1.29) | 1.38 (1.33-1.42) | 1.23 (1.19-1.27) | 1.36 (1.32-1.40) | 1.21 (1.18-1.25) |
|  | - | + | 2.12 (1.76-2.57) | 1.51 (1.24-1.83) | 1.91 (1.80-2.04) | 1.36(1.27-1.45) | 1.84 (1.76-1.92) | 1.32 (1.26-1.39) | 1.78 (1.71-1.85) | 1.27 (1.21-1.32) |
|  | + | + | 2.67 (2.39-2.97) | 1.88 (1.67-2.10) | 2.21 (2.12-2.31) | 1.54(1.46-1.61) | 2.18 (2.11-2.26) | 1.52 (1.46-1.58) | 2.16 (2.10-2.23) | 1.49 (1.44-1.55) |
| Liver | - | - | 1 | 1 | 1 | 1 | 1 | 1 | 1 | 1 |
|  | + | - | 1.33 (1.21-1.47) | 1.17 (1.06-1.29) | 1.28 (1.21-1.35) | 1.13(1.08-1.19) | 1.26 (1.21-1.32) | 1.12 (1.08-1.17) | 1.26 (1.21-1.31) | 1.12 (1.08-1.17) |
|  | - | + | 2.76 (2.19-3.48) | 1.99 (1.58-2.53) | 2.18 (2.01-2.35) | 1.55(1.42-1.68) | 1.95 (1.84-2.06) | 1.39 (1.30-1.48) | 1.87 (1.78-1.96) | 1.33 (1.26-1.40) |
|  | + | + | 2.53 (2.17-2.94) | 1.80 (1.53-2.12) | 2.23 (2.11-2.36) | 1.55(1.45-1.65) | 2.20 (2.11-2.30) | 1.52 (1.45-1.59) | 2,.12 (2.04-2.20) | 1.46 (1.39-1.52) |
| Neurological | - | - | 1 | 1 | 1 | 1 | 1 | 1 | 1 | 1 |
|  | + | - | 1.08 (1.02-1.15) | 0.96 (0.90-1.01) | 1.14 (1.10-1.17) | 1.01(0.98-1.04) | 1.12 (1.10-1.15) | 0.99 (0.97-1.02) | 1.10 (1.08-1.13) | 0.98 (0.95-0.99) |
|  | - | + | 1.87 (1.59-2.20) | 1.39 (1.17-1.64) | 1.68 (1.60-1.77) | 1.21(1.14-1.28) | 1.67 (1.62-1.73) | 1.18 (1.14-1.22) | 1.65 (1.60-1.70) | 1.14 (1.10-1.17) |
|  | + | + | 1.91 (1.73-2.10) | 1.39 (1.26-1.55) | 1.68 (1.62-1.74) | 1.17(1.12-1.22) | 1.63 (1.58-1.67) | 1.10 (1.07-1.14) | 1.58 (1.54-1.61) | 1.04 (1.01-1.07) |
| Psychological | - | - | 1 | 1 | 1 | 1 | 1 | 1 | 1 | 1 |
|  | + | - | 1.40 (1.33-1.48) | 1.25 (1.18-1.32) | 1.31 (1.27-1.35) | 1.18(1.15-1.22) | 1.30 (1.27-1.33) | 1.18 (1.15-1.21) | 1.30 (1.27-1.33) | 1.18 (1.16-1.21) |
|  | - | + | 2.39 (2.07-2.76) | 1.78 (1.53-2.05) | 1.78 (1.69-1.87) | 1.33(1.26-1.4) | 1.74 (1.68-1.81) | 1.30 (1.25-1.36) | 1.74 (1.68-1.79) | 1.30 (1.25-1.35) |
|  | + | + | 2.79 (2.56-3.03) | 2.05 (1.87-2.24) | 2.14 (2.07-2.21) | 1.57(1.51-1.63) | 2.03 (1.98-2.09) | 1.49 (1.44-1.53) | 2.00 (1.95-2.05) | 1.45 (1.41-1.50) |
| Renal | - | - | 1 | 1 | 1 | 1 | 1 | 1 | 1 | 1 |
|  | + | - | 1.05 (0.98-1.11) | 0.89 (0.83-0.95) | 0.97 (0.94-1.00) | 0.84(0.81-0.86) | 0.96 (0.94-0.98) | 0.83 (0.81-0.85) | 0.95 (0.93-0.98) | 0.83 (0.80-0.85) |
|  | - | + | 2.51 (2.16-2.93) | 1.73 (1.47-2.02) | 2.17 (2.08-2.27) | 1.45(1.39-1.52) | 1.99 (1.93-2.05) | 1.32 (1.28-1.37) | 1.93 (1.88-1.98) | 1.27 (1.24-1.31) |
|  | + | + | 2.52 (2.29-2.77) | 1.70 (1.54-1.89) | 1.97 (1.91-2.04) | 1.28(1.23-1.32) | 1.87 (1.83-1.92) | 1.19 (1.16-1.22) | 1.80 (1.76-1.84) | 1.13 (1.10-1.16) |
| Respiratory | - | - | 1 | 1 | 1 | 1 | 1 | 1 | 1 | 1 |
|  | + | - | 1.10 (1.03-1.19) | 0.94 (0.88-1.01) | 1.17 (1.13-1.21) | 1.02(0.99-1.06) | 1.16 (1.12-1.19) | 1.02 (0.99-1.05) | 1.16 (1.12-1.19) | 1.02 (0.99-1.05) |
|  | - | + | 2.01 (1.67-2.43) | 1.35 (1.11-1.63) | 1.85 (1.74-1.96) | 1.26(1.19-1.35) | 1.79 (1.71-1.87) | 1.23 (1.17-1.29) | 1.76 (1.70-1.83) | 1.22 (1.17-1.27) |
|  | + | + | 2.14 (1.90-2.40) | 1.42 (1.26-1.60) | 1.92 (1.84-2.01) | 1.28(1.22-1.34) | 1.89 (1.83-1.96) | 1.26 (1.21-1.30) | 1.88 (1.82-1.93) | 1.25 (1.21-1.29) |

aHR- Adjusted hazard ratio; uHR-unadjusted hazard ratio

\# adjusted for age at index date, sex, smoking, alcohol, propensity score, other comorbidities at index date, and other analgesics

CI- Confidence interval, HR- Hazard ratio, NSAIDs- Non-steroidal anti-inflammatory drugs; OA-Osteoarthritis.

Bold indicates significant p value <0.05 after adjusting for multiple testing problem.

# Table S2.5 Risk of comorbidities in analgesics (additionally adjusted for PPI)

|  |  |  | One year | | 5 years | | 10 years | | 22 years | |
| --- | --- | --- | --- | --- | --- | --- | --- | --- | --- | --- |
|  | OA | Drug | uHR(95% CI) | aHR(95% CI)^#^ | uHR(95% CI) | aHR(95% CI)^#^ | uHR(95% CI) | aHR(95% CI)^#^ | aHR(95% CI) | aHR(95% CI)^#^ |
| Cancer | - | - | 1 | 1 | 1 | 1 | 1 | 1 | 1 | 1 |
|  | + | - | 1.34 (1.23-1.46) | 1.34 (1.24-1.46) | 1.25 (1.20-1.30) | 1.26 (1.21-1.30) | 1.20 (1.17-1.24) | 1.21 (1.18-1.25) | 1.19 (1.16-1.23) | 1.20 (1.17-1.23) |
|  | - | + | 1.48 (1.08-2.03) | 1.48 (1.07-2.03) | 1.26 (1.15-1.37) | 1.23 (1.12-1.34) | 1.15 (1.09-1.21) | 1.12 (1.06-1.18) | 1.26 (1.20-1.32) | 1.24 (1.18-1.29) |
|  | + | + | 1.82 (1.57-2.10) | 1.81 (1.56-2.09) | 1.42 (1.35-1.50) | 1.38 (1.30-1.45) | 1.31 (1.26-1.36) | 1.27 (1.22-1.31) | 1.34 (1.29-1.39) | 1.31 (1.26-1.36) |
| Cardiovascular | - | - | 1 | 1 | 1 | 1 | 1 | 1 | 1 | 1 |
|  | + | - | 1.06 (1.02-1.10) | 1.00 (0.98-1.02) | 1.01 (0.99-1.03) | 1.03 (1.01-1.05) | 0.98 (0.97-1.00) | 1.07 (1.03-1.11) | 0.99 (0.97-1.00) | 1.00 (0.98-1.01) |
|  | - | + | 1.47 (1.27-1.69) | 1.07 (1.04-1.11) | 1.21 (1.16-1.27) | 1.16 (1.11-1.21) | 1.12 (1.09-1.16) | 1.42 (1.22-1.64) | 1.10 (1.07-1.13) | 1.06 (1.03-1.09) |
|  | + | + | 1.75 (1.64-1.88) | 1.14 (1.11-1.16) | 1.29 (1.26-1.33) | 1.23 (1.19-1.26) | 1.20 (1.18-1.23) | 1.68 (1.57-1.79) | 1.18 (1.15-1.20) | 1.11 (1.09-1.13) |
| Endocrine | - | - | 1 | 1 | 1 | 1 | 1 | 1 | 1 | 1 |
|  | + | - | 0.98 (0.94-1.03( | 0.99 (0.95-1.03) | 1.00 (0.98-1.03) | 1.02 (0.99-1.04) | 0.99 (0.98-1.01) | 1.01 (0.99-1.03) | 1.00 (0.98-1.01) | 1.01 (0.99-1.03) |
|  | - | + | 1.49 (1.26-1.75) | 1.44 (1.21-1.69) | 1.17 (1.11-1.23) | 1.12 (1.06-1.18) | 1.13 (1.10-1.18) | 1.08 (1.05-1.12) | 1.13 (1.09-1.16) | 1.08 (1.05-1.11) |
|  | + | + | 1.71 (1.58-1.85) | 1.64 (1.52-1.77) | 1.31 (1.27-1.35) | 1.24 (1.20-1.28) | 1.24 (1.22-1.28) | 1.18 (1.15-1.20) | 1.21 (1.19-1.24) | 1.15 (1.13-1.18) |
| Gastrointestinal | - | - | 1 | 1 | 1 | 1 | 1 | 1 | 1 | 1 |
|  | + | - | 1.19 (1.10-1.28) | 1.19 (1.10-1.29) | 1.26 (1.21-1.31) | 1.27 (1.22-1.32) | 1.25 (1.21-1.29) | 1.25 (1.21-1.29) | 1.23 (1.19-1.27) | 1.25 (1.21-1.29) |
|  | - | + | 1.16 (0.86-1.56) | 1.14 (0.84-1.55) | 1.54 (1.42-1.67) | 1.49 (1.37-1.61) | 1.32 (1.24-1.39) | 1.32 (1.25-1.39) | 1.33 (1.27-1.39) | 1.28 (1.22-1.35) |
|  | + | + | 1.94 (1.72-2.20) | 1.90 (1.67-2.15) | 1.73 (1.64-1.82) | 1.66 (1.58-1.75) | 1.58 (1.51-1.64) | 1.58 (1.52-1.64) | 1.60 (1.55-1.66) | 1.54 (1.48-1.59) |
| Liver | - | - | 1 | 1 | 1 | 1 | 1 | 1 | 1 | 1 |
|  | + | - | 1.12 (1.01-1.25) | 1.12 (1.01-1.24) | 1.12 (1.08-1.18) | 1.13 (1.08-1.19) | 1.14 (1.09-1.19) | 1.14 (1.10-1.19) | 1.14 (1.10-1.19) | 1.15 (1.07-1.19) |
|  | - | + | 1.39 (0.96-2.00) | 1.37 (0.95-1.99) | 1.18 (1.06-1.32) | 1.16 (1.04-1.29) | 1.16 (1.08-1.25) | 1.13 (1.05-1.21) | 1.10 (1.04-1.17) | 1.08 (1.01-1.14) |
|  | + | + | 1.31 (1.09-1.59) | 1.30 (1.08-1.58) | 1.28 (1.19-1.37) | 1.25 (1.16-1.33) | 1.30 (1.24-1.38) | 1.26 (1.20-1.33) | 1.25 (1.19-1.30) | 1.21 (1.16-1.27) |
| Neurological | - | - | 1 | 1 | 1 | 1 | 1 | 1 | 1 | 1 |
|  | + | - | 0.99 (0.93-1.06) | 1.00 (0.94-1.07) | 1.08 (1.04-1.11) | 1.09 (1.06-1.13) | 1.08 (1.05-1.10) | 1.09 (1.06-1.11) | 1.06 (1.04-1.09) | 1.07 (1.05-1.10) |
|  | - | + | 1.03 (0.78-1.36) | 0.98 (0.75-1.30) | 1.16 (1.08-1.25) | 1.11 (1.04-1.19) | 1.11 (1.06-1.16) | 1.07 (1.02-1.11) | 1.06 (1.02-1.09) | 1.02 (0.98-1.06) |
|  | + | + | 1.42 (1.26-1.59) | 1.34 (1.19-1.51) | 1.24 (1.19-1.30) | 1.18 (1.13-1.23) | 1.16 (1.12-1.20) | 1.11 (1.07-1.14) | 1.09 (1.07-1.13) | 1.05 (1.02-1.08) |
| Psychological | - | - | 1 | 1 | 1 | 1 | 1 | 1 | 1 | 1 |
|  | + | - | 1.13 (1.06-1.19) | 1.14 (1.08-1.21) | 1.11 (1.08-1.14) | 1.13 (1.09-1.16) | 1.10 (1.07-1.13) | 1.11 (1.09-1.14) | 1.10 (1.08-1.13) | 1.12 (1.09-1.15) |
|  | - | + | 1.42 (1.13-1.77) | 1.34 (1.07-1.67) | 1.24 (1.16-1.33) | 1.17 (1.09-1.26) | 1.20 (1.14-1.26) | 1.14 (1.08-1.19) | 1.17 (1.11-1.21) | 1.11 (1.06-1.15) |
|  | + | + | 2.05 (1.87-2.26) | 1.93 (1.75-2.12) | 1.49 (1.44-1.56) | 1.40 (1.34-1.46) | 1.39 (1.35-1.44) | 1.30 (1.26-1.35) | 1.34 (1.30-1.38) | 1.26 (1.22-1.30) |
| Renal | - | - | 1 | 1 | 1 | 1 | 1 | 1 | 1 | 1 |
|  | + | - | 0.98 (0.93-1.06) | 0.99 (0.93-1.06) | 0.98 (0.95-1.00) | 0.98 (0.95-1.01) | 0.98 (0.95-1.00) | 0.99 (0.96-1.01) | 0.97 (0.95-0.99) | 0.98 (0.96-1.00) |
|  | - | + | 1.81 (1.47-2.23) | 1.78 (1.44-2.19) | 1.28 (1.21-1.36) | 1.25 (1.17-1.32) | 1.16 (1.12-1.21) | 1.12 (1.08-1.17) | 1.14 (1.10-1.19) | 1.10 (1.07-1.14) |
|  | + | + | 1.38 (1.22-1.56) | 1.35 (1.20-1.52) | 1.18 (1.13-1.23) | 1.14 (1.09-1.19) | 1.17 (1.13-1.20) | 1.11 (1.08-1.14) | 1.14 (1.11-1.17) | 1.08 (1.06-1.11) |
| Respiratory | - | - | 1 | 1 | 1 | 1 | 1 | 1 | 1 | 1 |
|  | + | - | 0.95 (0.88-1.02) | 0.96 (0.89-1.03) | 1.06 (1.02-1.10) | 1.08 (1.04-1.12) | 1.06 (1.03-1.10) | 1.08 (1.05-1.11) | 1.06 (1.04-1.09) | 1.08 (1.05-1.11) |
|  | - | + | 1.24 (0.93-1.67) | 1.19 (0.89-1.59) | 1.19 (1.09-1.30) | 1.13 (1.04-1.23) | 1.17 (1.11-1.24) | 1.12 (1.06-1.19) | 1.14 (1.09-1.19) | 1.09 (1.04-1.14) |
|  | + | + | 1.59 (1.39-1.81) | 1.51 (1.32-1.72) | 1.33 (1.27-1.41) | 1.26 (1.19-1.33) | 1.29 (1.24-1.34) | 1.22 (1.17-1.27) | 1.24 (1.20-1.29) | 1.18 (1.13-1.21) |

aHR- Adjusted hazard ratio; uHR-unadjusted hazard ratio

\# adjusted for age at index date, sex, smoking, alcohol, propensity score, other comorbidities at index date, and other analgesics

CI- Confidence interval, HR- Hazard ratio, NSAIDs- Non-steroidal anti-inflammatory drugs; OA-Osteoarthritis.

Bold indicates significant p value <0.05 after adjusting for multiple testing problem.

# Table S2.6 Dose-response association of paracetamol use with comorbidities

|  | Cancer | Cardiovascular | Endocrine | Gastrointestinal | Renal | Liver | Neurological | Psychological | Respiratory |
| --- | --- | --- | --- | --- | --- | --- | --- | --- | --- |
|  | aHR (95% CI) ^#^ | aHR (95% CI) ^#^ | aHR (95% CI) ^#^ | aHR (95% CI) ^#^ | aHR (95% CI) ^#^ | aHR (95% CI) ^#^ | aHR (95% CI) ^#^ | aHR (95% CI) ^#^ | aHR (95% CI) ^#^ |
| Total |  |  |  |  |  |  |  |  |  |
| Q1 (Lowest) | 1 | 1 | 1 | 1 | 1 | 1 | 1 | 1 | 1 |
| Q2 | 1.23(1.12-1.35) | 1.28(1.18-1.38) | 1.20(1.11-1.29) | 1.25(1.11-1.4) | 1.23(1.15-1.33) | 1.43(1.25-1.64) | 1.21(1.12-1.32) | 1.35(1.21-1.50) | 1.28(1.14-1.43) |
| Q3 | 1.11(1.01-1.21) | 1.37(1.26-1.48) | 1.17(1.08-1.25) | 1.25(1.12-1.39) | 1.15(1.07-1.24) | 1.21(1.06-1.39) | 1.20(1.11-1.30) | 1.39(1.25-1.54) | 1.3(1.17-1.46) |
| Q4 | 0.99(0.90-1.09) | 1.54(1.43-1.66) | 1.20(1.12-1.29) | 1.38(1.24-1.54) | 1.26(1.17-1.34) | 1.36(1.19-1.55) | 1.17(1.09-1.27) | 1.36(1.23-1.50) | 1.46(1.31-1.62) |
| Q5 (Highest) | 1.75(1.60-1.91) | 3.56(3.31-3.83) | 2.89(2.70-3.10) | 3.09(2.79-3.41) | 2.68(2.51-2.86) | 2.48(2.13-2.86) | 2.32(2.16-2.50) | 3.17(2.88-3.48) | 3.47(3.14-3.84) |
| Osteoarthritis |  |  |  |  |  |  |  |  |  |
| Q1 (Lowest) | 1 | 1 | 1 | 1 | 1 | 1 | 1 | 1 | 1 |
| Q2 | 1.22(1.09-1.37) | 1.31(1.19-1.46) | 1.19(1.09-1.32) | 1.22(1.06-1.40) | 1.24(1.13-1.37) | 1.34(1.13-1.59) | 1.23(1.1-1.37) | 1.31(1.14-1.50) | 1.24(1.07-1.44) |
| Q3 | 1.10(0.98-1.13) | 1.42(1.28-1.56) | 1.16(1.05-1.27) | 1.15(1.00-1.32) | 1.19(1.08-1.30) | 1.11(0.94-1.32) | 1.20(1.08-1.33) | 1.39(1.22-1.59) | 1.40(1.21-1.62) |
| Q4 | 0.96(0.86-1.07) | 1.59(1.45-1.75) | 1.24(1.14-1.36) | 1.32(1.16-1.51) | 1.28(1.17-1.40) | 1.30(1.10-1.53) | 1.20(1.08-1.33) | 1.34(1.18-1.52) | 1.39(1.21-1.60) |
| Q5 (Highest) | 1.70(1.53-1.89) | 3.72(3.39-4.08) | 3.04(2.79-3.31) | 3.07(2.71-3.48) | 2.79(2.56-3.04) | 2.95(2.54-3.44) | 2.57(2.33-2.83) | 3.31(2.94-3.74) | 3.48(3.05-3.96) |
| Non-Osteoarthritis |  |  |  |  |  |  |  |  |  |
| Q1 (Lowest) | 1 | 1 | 1 | 1 | 1 | 1 | 1 | 1 | 1 |
| Q2 | 1.20(1.03-1.40) | 1.21(1.06-1.38) | 1.20(1.06-1.36) | 1.25(1.03-1.52) | 1.24(1.10-1.38) | 1.58(1.26-1.98) | 1.21(1.07-1.37) | 1.42(1.20-1.67) | 1.35(1.12-1.60) |
| Q3 | 1.07(0.92-1.25) | 1.26(1.11-1.44) | 1.17(1.04-1.32) | 1.38(1.14-1.67) | 1.11(0.99-1.13) | 1.37(1.09-1.71) | 1.23(1.09-1.39) | 1.35(1.14-1.59) | 1.12(0.93-1.13) |
| Q4 | 1.02(0.89-1.19) | 1.43(1.26-1.62) | 1.09(0.97-1.23) | 1.39(1.15-1.67) | 1.22(1.10-1.36) | 1.40(1.11-1.74) | 1.16(1.03-1.29) | 1.38(1.18-1.63) | 1.59(1.34-1.89) |
| Q5 (Highest) | 1.80(1.55-2.10) | 3.26(2.88-3.67) | 2.55(2.27-2.86) | 2.86(2.40-3.41) | 2.54(2.29-2.81) | 2.84(2.29-3.52) | 1.95(1.74-2.20) | 2.83(2.42-3.30) | 3.49(2.96-4.10) |

aHR- Adjusted hazard ratio; uHR-unadjusted hazard ratio

\# adjusted for age at index date, sex, smoking, alcohol, propensity score, other comorbidities at index date, and other analgesics

CI- Confidence interval, HR- Hazard ratio, NSAIDs- Non-steroidal anti-inflammatory drugs; OA-Osteoarthritis.

Bold indicates significant p value <0.05 after adjusting for multiple testing problem.

# Table S2.7 Dose-response association of NSAID use with comorbidities

|  | Cancer | Cardiovascular | Endocrine | Gastrointestinal | Renal | Liver | Neurological | Psychological | Respiratory |
| --- | --- | --- | --- | --- | --- | --- | --- | --- | --- |
|  | aHR (95% CI) ^#^ | aHR (95% CI) ^#^ | aHR (95% CI) ^#^ | aHR (95% CI) ^#^ | aHR (95% CI) ^#^ | aHR (95% CI) ^#^ | aHR (95% CI) ^#^ | aHR (95% CI) ^#^ | aHR (95% CI) ^#^ |
| Total |  |  |  |  |  |  |  |  |  |
| Q1 (Lowest) | 1 | 1 | 1 | 1 | 1 | 1 | 1 | 1 | 1 |
| Q2 | 1.39(1.29-1.51) | 1.45(1.38-1.53) | 1.36(1.29-1.43) | 1.55(1.42-1.69) | 1.48(1.39-1.58) | 1.40(1.28-1.56) | 1.39(1.29-1.48) | 1.40(1.29-1.52) | 1.45(1.33-1.57) |
| Q3 | 1.58(1.46-1.70) | 1.76(1.67-1.85) | 1.6(1.52-1.68) | 1.68(1.55-1.83) | 1.82(1.72-1.94) | 1.52(1.37-1.69) | 1.61(1.51-1.72) | 1.56(1.44-1.68) | 1.52(1.39-1.65) |
| Q4 | 1.52(1.41-1.65) | 1.97(1.87-2.07) | 1.75(1.66-1.83) | 1.69(1.56-1.84) | 2.16(2.04-2.30) | 1.59(1.43-1.77) | 1.66(1.56-1.78) | 1.58(1.46-1.70) | 1.66(1.53-1.80) |
| Q5 (Highest) | 2.69(2.49-2.91) | 4.63(4.41-4.86) | 3.7(3.52-3.89) | 3.67(3.39-3.98) | 3.55(3.29-3.82) | 3.02(2.72-3.34) | 3.07(2.88-3.28) | 3.28(3.05-3.53) | 3.57(3.30-3.87) |
| Osteoarthritis |  |  |  |  |  |  |  |  |  |
| Q1 (Lowest) | 1 | 1 | 1 | 1 | 1 | 1 | 1 | 1 | 1 |
| Q2 | 1.28(1.15-1.44) | 1.33(1.24-1.43) | 1.27(1.18-1.37) | 1.39(1.24-1.56) | 1.35(1.24-1.47) | 1.27(1.10-1.47) | 1.40(1.28-1.55) | 1.25(1.12-1.39) | 1.33(1.18-1.50) |
| Q3 | 1.51(1.36-1.68) | 1.66(1.55-1.78) | 1.53(1.42-1.64) | 1.53(1.37-1.71) | 1.7(1.57-1.85) | 1.39(1.21-1.60) | 1.66(1.51-1.82) | 1.36(1.22-1.51) | 1.43(1.27-1.60) |
| Q4 | 1.49(1.35-1.66) | 1.93(1.81-2.06) | 1.74(1.62-1.86) | 1.56(1.40-1.74) | 2.12(1.96-2.30) | 1.48(1.29-1.70) | 1.74(1.59-1.91) | 1.43(1.29-1.58) | 1.63(1.46-1.83) |
| Q5 (Highest) | 2.71(2.45-2.99) | 4.75(4.44-5.07) | 3.84(3.59-4.10) | 3.50(3.15-3.89) | 3.48(3.14-3.85) | 2.89(2.53-3.30) | 3.28(3.00-3.59) | 3.15(2.86-3.48) | 3.56(3.20-3.97) |
| Non-Osteoarthritis |  |  |  |  |  |  |  |  |  |
| Q1 (Lowest) | 1 | 1 | 1 | 1 | 1 | 1 | 1 | 1 | 1 |
| Q2 | 1.54(1.37-1.73) | 1.68(1.56-1.80) | 1.56(1.44-1.68) | 1.76(1.55-2.00) | 1.72(1.57-1.88) | 1.59(1.35-1.87) | 1.42(1.29-1.57) | 1.64(1.45-1.84) | 1.63(1.44-1.84) |
| Q3 | 1.67(1.47-1.89) | 2.07(1.92-2.24) | 1.88(1.73-2.03) | 1.92(1.68-2.20) | 2.17(1.98-2.39) | 1.78(1.50-2.11) | 1.68(1.51-1.87) | 2.02(1.78-2.28) | 1.74(1.53-1.99) |
| Q4 | 1.52(1.32-1.75) | 2.17(1.99-2.35) | 1.88(1.73-2.04) | 1.87(1.61-2.16) | 2.32(2.10-2.56) | 1.77(1.47-2.12) | 1.69(1.51-1.89) | 1.93(1.70-2.19) | 1.73(1.50-1.99) |
| Q5 (Highest) | 2.44(2.12-2.80) | 4.20(3.87-4.57) | 3.31(3.03-3.61) | 3.46(3.01-3.99) | 3.82(3.38-4.30) | 2.97(2.48-3.55) | 2.90(2.59-3.24) | 3.02(2.66-3.44) | 3.45(3.01-3.95) |

aHR- Adjusted hazard ratio; uHR-unadjusted hazard ratio

\# adjusted for age at index date, sex, smoking, alcohol, propensity score, other comorbidities at index date, and other analgesics

CI- Confidence interval, HR- Hazard ratio, NSAIDs- Non-steroidal anti-inflammatory drugs; OA-Osteoarthritis.

Bold indicates significant p value <0.05 after adjusting for multiple testing problem.

# Table S2.8 Dose-response association of opioid use with comorbidities

|  | Cancer | Cardiovascular | Endocrine | Gastrointestinal | Renal | Liver | Neurological | Psychological | Respiratory |
| --- | --- | --- | --- | --- | --- | --- | --- | --- | --- |
|  | aHR (95% CI)^#^ | aHR (95% CI)^#^ | aHR (95% CI)^#^ | aHR (95% CI)^#^ | aHR (95% CI)^#^ | aHR (95% CI)^#^ | aHR (95% CI)^#^ | aHR (95% CI)^#^ | aHR (95% CI)^#^ |
| Total |  |  |  |  |  |  |  |  |  |
| Q1 (Lowest) | 1 | 1 | 1 | 1 | 1 | 1 | 1 | 1 | 1 |
| Q2 | 1.55(1.45-1.66) | 1.45(1.37-1.52) | 1.39(1.32-1.46) | 1.36(1.26-1.47) | 1.36(1.27-1.47) | 1.58(1.38-1.81) | 1.31(1.19-1.44) | 1.85(1.68-2.05) | 1.30(1.16-1.46) |
| Q3 | 1.57(1.47-1.68) | 1.62(1.54-1.70) | 1.45(1.38-1.52) | 1.5(1.39-1.63) | 1.39(1.30-1.49) | 1.59(1.41-1.79) | 1.38(1.28-1.49) | 1.82(1.67-1.98) | 1.33(1.21-1.47) |
| Q4 | 1.50(1.40-1.67) | 1.89(1.80-1.98) | 1.59(1.51-1.67) | 1.54(1.42-1.66) | 1.42(1.34-1.51) | 1.63(1.45-1.82) | 1.39(1.30-1.50) | 1.96(1.82-2.12) | 1.41(1.29-1.54) |
| Q5 (Highest) | 2.86(2.68-3.06) | 4.62(4.40-4.85) | 4.17(3.98-4.37) | 4.12(3.82-4.43) | 2.71(2.58-2.85) | 2.29(2.03-2.59) | 1.86(1.71-2.03) | 3.54(3.28-3.81) | 2.52(2.36-2.70) |
| Osteoarthritis |  |  |  |  |  |  |  |  |  |
| Q1 (Lowest) | 1 | 1 | 1 | 1 | 1 | 1 | 1 | 1 | 1 |
| Q2 | 1.62(1.48-1.79) | 1.44(1.34-1.54) | 1.34(1.25-1.44) | 1.30(1.17-1.44) | 1.27(1.15-1.40) | 1.51(1.27-1.80) | 1.29(1.14-1.46) | 1.77(1.55-2.00) | 1.29(1.11-1.49) |
| Q3 | 1.69(1.55-1.86) | 1.68(1.57-1.80) | 1.50(1.40-1.60) | 1.38(1.25-1.53) | 1.38(1.27-1.50) | 1.50(1.29-1.75) | 1.31(1.19-1.44) | 1.78(1.60-1.98) | 1.32(1.17-1.49) |
| Q4 | 1.60(1.46-1.75) | 1.98(1.85-2.11) | 1.65(1.54-1.76) | 1.45(1.31-1.60) | 1.44(1.34-1.55) | 1.55(1.35-1.77) | 1.38(1.26-1.51) | 1.80(1.63-1.98) | 1.49(1.33-1.65) |
| Q5 (Highest) | 3.05(2.79-3.33) | 4.96(4.65-5.30) | 4.46(4.18-4.76) | 4.01(3.64-4.42) | 2.82(2.66-2.99) | 2.39(2.07-2.74) | 2.01(1.81-2.22) | 3.46(3.17-3.78) | 2.58(2.38-2.80) |
| Non-Osteoarthritis |  |  |  |  |  |  |  |  |  |
| Q1 (Lowest) | 1 | 1 | 1 | 1 | 1 | 1 | 1 | 1 | 1 |
| Q2 | 1.53(1.39-1.69) | 1.50(1.40-1.62) | 1.50(1.39-1.61) | 1.42(1.26-1.61) | 1.50(1.35-1.68) | 1.67(1.35-2.06) | 1.41(1.24-1.62) | 1.97(1.68-2.30) | 1.32(1.09-1.59) |
| Q3 | 1.44(1.30-1.60) | 1.59(1.47-1.71) | 1.41(1.31-1.53) | 1.69(1.49-1.90) | 1.43(1.29-1.59) | 1.77(1.46-2.16) | 1.49(1.33-1.68) | 1.87(1.62-2.17) | 1.34(1.14-1.57) |
| Q4 | 1.42(1.28-1.58) | 1.82(1.68-1.97) | 1.50(1.39-1.63) | 1.62(1.43-1.83) | 1.43(1.29-1.59) | 1.89(1.55-2.29) | 1.44(1.27-1.63) | 2.33(2.05-2.66) | 1.28(1.09-1.51) |
| Q5 (Highest) | 2.72(2.45-3.02) | 4.11(3.80-4.44) | 3.67(3.40-3.95) | 3.93(3.50-4.42) | 2.57(2.35-2.82) | 2.26(1.76-2.84) | 1.62(1.34-1.91) | 3.64(3.15-4.18) | 2.52(2.22-2.86) |

aHR- Adjusted hazard ratio; uHR-unadjusted hazard ratio

\# adjusted for age at index date, sex, smoking, alcohol, propensity score, other comorbidities at index date, and other analgesics

CI- Confidence interval, HR- Hazard ratio, NSAIDs- Non-steroidal anti-inflammatory drugs; OA-Osteoarthritis.

Bold indicates significant p value <0.05 after adjusting for multiple testing problem.

# Figure S2.1 Cumulative hazard of risk of comorbidities among paracetamol users

# Figure S2.2 Cumulative hazard of risk of comorbidities among NSAID users

# Figure S2.3 Cumulative hazard of risk of comorbidities among opioid users

# Table S2.9 Classification of NSAIDs

| **COX inhibition classes** | **Name of the drugs/composition** |
| --- | --- |
| COX 1 selective | Ketoprofen , Ketorolac, Fenbufen |
| Non-selective | Diclofenac , Ibuprofen , Naproxen , Dexibuprofen , Nabumetone , Indomethacin , Piroxicam,  Mefenamic acid, Aceclofenac, Flurbiprofen, Tenoxicam, Acemetacin |
| COX 2 selective | Celecoxib , Rofecoxib , Parecoxib , Etoricoxib , Lumiracoxib , Meloxicam, Etodolac |

# Table S2.10 Types of NSAID and the risk of comorbidities

|  | Cancer | Cardiovascular | Endocrine | Gastrointestinal | Renal | Liver | Neurological | Psychological | Respiratory |
| --- | --- | --- | --- | --- | --- | --- | --- | --- | --- |
|  | aHR (95% CI) | aHR (95% CI) | aHR (95% CI) | aHR (95% CI) | aHR (95% CI) | aHR (95% CI) | aHR (95% CI) | aHR (95% CI) | aHR (95% CI) |
| Non users | Reference | Reference | Reference | Reference | Reference | Reference | Reference | Reference | Reference |
| Non-selective | 0.99 [0.86-1.15] | 1.25 [1.20-1.30] | 1.15 [1.10-1.19] | 0.95 [0.89-1.02] | 1.19 [1.13-1.26] | 0.93 [0.85-1.01] | 0.91 [0.86-0.97] | 1.21 [1.15-1.28] | 1.01 [0.95-1.08] |
| COX-1 | 0.96 [0.19-4.69] | 1.17 [0.78-1.77] | 0.75 [0.44-1.29] | 1.15 [0.58-2.31] | 1.05 [0.63-1.75] | 1.10 [0.41-2.96] | 0.74 [0.36-1.53] | 1.05 [0.55-1.98] | 1.12 [0.60-2.09] |
| COX-2 | 1.65 [1.08-2.52] | 1.34 [1.25-1.44] | 1.13 [1.04-1.21] | 1.05 [0.93-1.18] | 1.25 [1.14-1.37] | 0.88 [0.74-1.05] | 0.85 [0.75-0.95] | 1.21 [1.09-1.34] | 1.06 [0.94-1.19] |

aHR- Adjusted hazard ratio; uHR-unadjusted hazard ratio

\# adjusted for age at index date, sex, smoking, alcohol, propensity score, other comorbidities at index date, and other analgesics

CI- Confidence interval, HR- Hazard ratio, NSAIDs- Non-steroidal anti-inflammatory drugs; OA-Osteoarthritis.

Bold indicates significant p value <0.05 after adjusting for multiple testing problem.
